# Matching-adjusted indirect comparison of asciminib versus other treatments in chronic-phase chronic myeloid leukemia after failure of two prior tyrosine kinase inhibitors

**DOI:** 10.1007/s00432-022-04562-5

**Published:** 2023-01-28

**Authors:** Ehab Atallah, Michael J. Mauro, Andreas Hochhaus, Carla Boquimpani, Yosuke Minami, Vikalp Kumar Maheshwari, Lovneet Saini, Regina Corbin, Delphine Réa

**Affiliations:** 1 Medical College of Wisconsin, Milwaukee, WI USA; 2 Memorial Sloan-Kettering Cancer Center, New York, NY USA; 3 Universitätsklinikum Jena, Jena, Germany; 4 HEMORIO, State Institute of Hematology Arthur de Siquiera Cavalcanti, Rio de Janeiro, Brazil; 5 Oncoclínica Centro de Tratamento Oncológico, Rio de Janeiro, Brazil; 6 National Cancer Center Hospital East, Kashiwa, Japan; 7 Novartis Healthcare Pvt. Ltd, Hyderabad, India; 8 Novartis Services Inc., East Hanover, NJ USA; 9 Adult Hematology Hôpital Saint-Louis and FiLMC, Paris, France

**Keywords:** Chronic myeloid leukemia, Asciminib, ASCEMBL, Tyrosine kinase inhibitors, Indirect treatment comparison

## Abstract

**Purpose:**

The current standard of care for chronic-phase chronic myeloid leukemia (CP-CML) is tyrosine kinase inhibitors (TKIs). Treatment recommendations are unclear for CP-CML failing ≥ 2 lines of treatment, partly due to the paucity of head-to-head trials evaluating TKIs. Thus, matching-adjusted indirect comparisons (MAICs) were conducted to compare asciminib with competing TKIs in third- or later line (≥ 3L) CP-CML.

**Methods:**

Individual patient-level data for asciminib (ASCEMBL; follow-up: ≥ 48 weeks) and published aggregate data for comparator TKIs (ponatinib, nilotinib, and dasatinib) informed the analyses. Major molecular response (MMR), complete cytogenetic response (CCyR), and time to treatment discontinuation (TTD) were assessed, where feasible.

**Results:**

Asciminib was associated with statistically significant improvements in MMR by 6 (relative risk [RR]: 1.55; 95% confidence interval [CI]: 1.02, 2.36) and 12 months (RR: 1.48; 95% CI: 1.03, 2.14) vs ponatinib. For CCyR, the results vs ponatinib were similar by 6 (RR: 1.11; 95% CI: 0.81, 1.52) and 12 months (RR: 0.97; 95% CI: 0.73, 1.28). Asciminib was associated with improvements in MMR by 6 months vs dasatinib but with a CI overlapping one (RR 1.52; 95% CI: 0.66, 3.53). Asciminib was associated with statistically significant improvements in CCyR by 6 (RR: 3.57; 95% CI: 1.42, 8.98) and 12 months (RR: 2.03; 95% CI: 1.12, 3.67) vs nilotinib/dasatinib. Median TTD was unreached for asciminib in ASCEMBL. However, post-adjustment asciminib implied prolonged TTD vs nilotinib and dasatinib, but not vs ponatinib.

**Conclusion:**

These analyses demonstrate favorable outcomes with asciminib versus competing TKIs, highlighting its therapeutic potential in ≥ 3L CP-CML.

**Supplementary Information:**

The online version contains supplementary material available at 10.1007/s00432-022-04562-5.

## Introduction

Chronic myeloid leukemia (CML) is characterized by abnormal uncontrolled proliferation of myeloid cells (Hehlmann et al. 2007; Cortes and Lang 2021). In majority of cases, its pathogenesis is due to unfaithful DNA repair resulting in the formation of an abnormal Philadelphia chromosome, the reciprocal translocation between chromosomes 9 and 22. (Hehlmann et al. 2007; Rohrbacher and Hasford 2018; Cortes and Lang 2021). At diagnosis, approximately 90–95% of patients with CML are in the chronic phase (CP) (Jabbour and Kantarjian 2020). Untreated CML progresses from the CP to the advanced phases, which comprise the accelerated phase (AP) and the blastic phase (BP) (Hochhaus et al. 2017; NCCN 2021). The estimated annual incidence of CML ranges from approximately 1/100,000 in Europe (Hoffmann et al. 2014) to 1.9/100,000 in the United States (US) (ACS 2018; SEER 2021).

Prior to the availability of ABL kinase inhibitors, median survival among untreated CP-CML patients ranged from 3 to 5 years. The advent of tyrosine kinase inhibitors (TKIs) has since improved median survival in CP-CML such that it was comparable to that of the general population; this improvement was especially seen in patients who at least achieved a complete cytogenetic response (CCyR) (Kantarjian et al. 1993; Sasaki et al. 2015). However, this improvement has not been demonstrated in all patient subgroups (Bower et al. 2016). Either the first-generation (imatinib) or a second-generation (2G; nilotinib, dasatinib, bosutinib) TKI is recommended as the frontline therapy for CP-CML by the National Comprehensive Cancer Network (NCCN) guidelines and European LeukemiaNet (ELN) recommendations (Hochhaus et al. 2017; Hochhaus et al. 2020a, b; NCCN 2021). However, almost 40% of patients require change to another treatment within 5 years of targeted therapy (Cortes et al. 2016; Hochhaus et al. 2016; Brümmendorf et al. 2020; Kantarjian et al. 2021). In addition, only 50% of patients resistant to imatinib in first line achieve complete cytogenetic remission (CCyR) with second-line (2L) therapy (Hochhaus et al. 2011; Jabbour and Kantarjian 2016). Patients whose disease fails to respond to multiple TKIs are reportedly susceptible to show lack of durable response with an alternative 2G TKI as third- or later line (≥ 3L) therapy (Garg et al. 2009; Ibrahim et al. 2010; Ribeiro et al. 2015; Levy et al. 2019). Although ponatinib is known to be a potent choice for highly pretreated patients, its use is associated with life-threatening cardiovascular events requiring thorough monitoring and addition of treatment for risk factors, as well as potential dose reductions (Garcia-Gutierrez and Hernandez-Boluda 2019; Mauro 2021).

For patients receiving ≥ 3L therapy, the treatment options include alternative TKIs that might not have been previously used, such as nilotinib, dasatinib, bosutinib, ponatinib, and asciminib. Allogeneic stem cell transplantation (allo-SCT) is a potentially curative therapeutic modality in selected patients with either CP- or AP-CML, often as a treatment of last resort, with the ability to deliver long-term survival (Cortes and Lang 2021). However, it is associated with significant morbidity and mortality. Current guidelines and treatment recommendations are unclear in defining the optimal treatment pathway in patients receiving ≥ 3L therapy, and treatment options in later lines become limited due to resistance or intolerance to multiple TKIs (Hochhaus et al. 2017; Hochhaus et al. 2020a, b; NCCN 2021). Asciminib, an inhibitor specifically targeting the ABL myristoyl pocket (STAMP) (Wylie et al. 2017; Manley et al. 2020; Novartis 2021), is promising for use as a targeted agent in patients with CP-CML. While exhibiting specific action on the ABL family of kinases, it remains inactive against other kinases. Due to limited off-target activity, the safety profile of asciminib is very promising, compared to existing TKIs (Mauro 2021). Asciminib was given recent FDA approval based on its superior efficacy and favorable safety profile compared with bosutinib after 24 weeks of therapy in the ASCEMBL (NCT03106779) trial (Novartis 2021).

ASCEMBL is an open-label, randomized, phase III trial comparing asciminib with bosutinib as a ≥ 3L therapy in patients with CP-CML, where the primary endpoint was to assess the superiority in achieving major molecular response (MMR; BCR::ABL1 ≤ 0.1%) at week 24 (Rea et al. 2021). The trial met the primary objective with an MMR rate of 25.5% vs 13.2% with asciminib vs bosutinib, respectively (Rea et al. 2021). The between-arm difference in MMR at 24 and 48 weeks was 12.2% and 16.1%, respectively, after adjustment for major cytogenetic response status at baseline. With fewer grade ≥ 3 adverse events (AEs; 50.6% vs 60.5%) and treatment discontinuation due to AEs (5.8% vs 21.1%), asciminib showed promising safety along with favorable efficacy results (Rea et al. 2021).

ASCEMBL is the first and only head-to-head randomized controlled trial (RCT) in patients with CP-CML previously treated with multiple TKIs. Although RCTs facilitating direct or head-to-head comparisons provide the most valid treatment effectiveness estimates, there is often a paucity of RCTs evaluating each competing intervention in a given therapeutic area. RCTs are often time-consuming, costly, and sometimes impractical to conduct especially in settings where there is a rapidly changing treatment landscape populated by several, or even numerous competing interventions. Moreover, RCTs often compare interventions to a standard of care or placebo. Consequently, it is common for there to be an absence of any direct comparisons between a new treatment and one or more relevant competitor interventions. Given that evidence-based healthcare decision-making requires the assessment of all alternative interventions, there is a need to conduct a comprehensive analysis that includes all relevant interventions despite the constraints of RCTs.

In the absence of direct head-to-head trials comparing asciminib with all other competing interventions for ≥ 3L CP-CML, indirect treatment comparisons (ITCs) are required to estimate the relative treatment effect between asciminib and other TKIs. ITCs involve comparisons of non-randomized treatment groups and are akin to observational studies and subject to important limitations. In particular, cross-study differences in patients’ baseline characteristics or other study characteristic differences can bias indirect comparative effectiveness results. These limitations are difficult to address using only published aggregate data (AD). However, indirect treatment comparisons such as matching-adjusted indirect comparisons (MAICs) use statistical methods to simulate a direct comparison of two therapies (Signorovitch et al. 2012; Phillippo et al. 2016, 2018) by matching individual patient-level data (IPD) from one trial to published AD from another trial (Dias et al. 2013). As such, MAICs that leverage IPD may generate the best comparative evidence available. The availability of IPD from ASCEMBL and published AD from comparator studies permitted adjustment of cross-study differences in baseline characteristics and reduced other study characteristic differences to allow the comparisons of more clinically similar studies. In the United Kingdom (UK), MAICs have been used in over 20 successful drug reimbursement evaluations and are included in methodological guidance for indirect comparisons issued by the National Institute of Health and Care Excellence (NICE) (Ivanescu et al. 2017).

The objective of this study was to conduct an MAIC, estimating the relative efficacy of asciminib compared with TKIs other than bosutinib, commonly used in treating ≥ 3L CP-CML patients, to inform clinical practice in the absence of head-to-head RCTs.

## Methods

### Study selection

A clinical systematic literature review (SLR) was conducted by searching the EMBASE®, MEDLINE®, and CENTRAL databases through OVID® to identify all relevant publications, available from database inception to May 2021, reporting results in adult (aged ≥ 18 years) patients with CP-CML who had received ≥ 2 TKIs prior to study entry. The SLR was performed and reported in accordance with the Preferred Reporting Items for Systematic Reviews and Meta-Analyses (PRISMA) guidelines (Online Resource 1).

The identified studies were shortlisted for the MAIC analyses if they either reported the baseline characteristics for the subgroup containing patients of interest for the current analysis or the baseline characteristics for a group of participants where ≥ 75% of patients matched the target population (prior treatment with ≥ 2 TKIs and absence of the T315I mutation), with the assumption that the outcomes in the overall patient population will represent the outcomes for the patients of interest for this analysis. Interventions reported among the identified studies included: ponatinib, dasatinib, nilotinib, and omacetaxine. Given that TKIs are the global standard of care for ≥ 3L CP-CML, the MAIC analyses only included studies investigating TKIs (i.e., ponatinib, nilotinib, and dasatinib). Detailed eligibility criteria used for identifying the studies eligible for the analysis are presented in Online Resource 2. Studies not included in the MAIC are listed in Online Resource 3 Reference source not found along with the reason for exclusion.

A summary of the six unique studies that met the eligibility criteria to inform the MAIC analyses is presented in Table 1.

**Table 1:** Summary of study and patient characteristics of the included studies

Study	Study design	Intervention	*N*	Age, years Median (range)	ECOG PS	Exposure to prior regimens	T315I mutation	CCyR at study entry	Study follow-up or treatment duration
ASCEMBL (NCT03106779)	Open-label phase III RCT	Asciminib: 20 mg or 40 mg BID; orally	157	52 (24–83)	0–2	≥ 2 prior lines of TKI Failure or intolerance to the last previous TKI therapy at the time of screening Those with prior allo-SCT were excluded	Excluded	Allowed	IPD available for MAIC: data cutoff January 6, 2021, for patients with follow-up of ≥ 48 weeks
		Bosutinib: 100 mg or 500 mg QD; orally	76	52 (19–77)					
PACE (NCT01207440)	Phase II single-arm trial	Ponatinib: 45 mg QD; orally	203	61 (18–94)	0–2	Resistant or intolerant to dasatinib or nilotinib	Allowed	Excluded	Median follow-up: 56.8 months (range: 0.1–73.1) Median treatment duration: 32.1 months (range: 0.1–73.0)
Giles et al. (2010)	Phase II single-arm trial	Nilotinib: 400 mg BID; orally	39	62 (34–78)	–	Resistance to or intolerance of imatinib Failure to respond to dasatinib	Allowed	–	Median follow-up: 12 months Median treatment duration: 11 months (range: < 1.0–29.2)
Tan et al. (2019)	Single-center retrospective chart review	Dasatinib: 100 mg QD; orally	24	50 (34–68)	–	Failure of imatinib, AND Failure of nilotinib	Excluded	–	Study duration: 12 months
Rossi et al. (2013)	Multicenter prospective observational study	Nilotinib: 400 mg BID; orally	48	60 (33–80)	–	Failed imatinib, AND Failed dasatinib or nilotinib	Allowed	–	Median follow-up: 14 months (range: 2–37)
		Dasatinib: 100 mg QD; orally	34	60 (43–85)					
Ibrahim et al. (2010)	Single-center prospective observational study	Nilotinib/dasatinib	26	64	–	Failed imatinib, AND Failed dasatinib or nilotinib	Excluded	–	Median follow-up: 21.5 months (range: 6–46.5)

*Allo-SCT* allogeneic stem cell transplantation; *BID* twice daily; *CCyR* complete cytogenetic response; *ECOG PS* Eastern Cooperative Oncology Group Performance Status; *MAIC* matching-adjusted indirect comparison; *QD* once daily; *RCT* randomized controlled trial; *TKI* tyrosine kinase inhibitor

### Outcomes

Five outcomes were assessed in this analysis: MMR rate by 6 and by 12 months, CCyR rate by 6 and by 12 months, time to MMR by 6 and by 12 months, time to CCyR by 6 and by 12 months, and TTD. Of note, time to MMR and CCyR curves were reported for ponatinib only, which were digitized using WebPlotDigitizer (v4.5) to retrieve the relevant data points. TTD Kaplan–Meier (KM) curves were not available for any comparator, hence the median treatment duration reported for each comparator was used as an alternative and included in the analysis for comparing the median TTD reported for asciminib in the ASCEMBL trial.

Comorbidities and drug safety profiles are of importance when choosing the TKI upon starting treatment and disease management. Hence, the intent was to also compare the safety outcomes across current ≥ 3L CP-CML treatment options. However, the use of the MAIC method to compare the safety outcomes across trials was not feasible due to the varying definitions of safety outcomes and classification of adverse events (AEs). Alternatively, a naïve comparison was undertaken (Levy et al. 2019). For the naïve comparison of safety outcomes, the reported rates of any-cause AEs, treatment-related AEs, serious AEs, treatment discontinuation due to AEs, and treatment-related deaths with asciminib and other ≥ 3L TKIs were compared.

### Statistical analyses

## Matching-adjusted indirect comparison

Given the lack of a common comparator, an unanchored MAIC was used to estimate the relative treatment effect of asciminib vs other ≥ 3L TKIs by leveraging IPD from ASCEMBL and published AD from comparator studies. Additional details of the MAIC methodology are provided in Online Resource 4.

The ASCEMBL population was adjusted to match the eligibility criteria and distribution of prognostic factors in each of the comparator studies. Patients in ASCEMBL who did not fulfill the eligibility criteria of the comparator study were removed to better align the two populations (Table 2). Patients from ASCEMBL who satisfied the eligibility criteria of each comparator study were then reweighted to adjust for imbalances in baseline characteristics of prognostic significance. A form of propensity score weighting was used, in which patients in ASCEMBL were weighted by the inverse odds of being in that group compared to the other group (derived from the competitor study for which only AD was available). Weights were based on a generalized method-of-moments propensity score algorithm, which guaranteed a close balancing of covariates between the ASCEMBL and comparator populations. Results of the ASCEMBL study were re-analyzed using the weighted patient-level data set. Treatment outcomes were then compared across balanced study populations. To quantify the overlap between the two study populations, the effective sample size (ESS) was calculated to reflect the impact of weighting on the available information in the IPD. The ESS is the number of non-weighted patients that would produce a treatment effect estimate with the same precision as the reweighted sample estimate. Since these MAIC analyses provided an unanchored indirect comparison due to the lack of a common comparator arm in each comparison, all treatment effect modifiers and prognostic variables should be adjusted to ensure balance and reduce bias (Phillippo et al. 2018).

**Table 2:** Summary of results of matching performed for the asciminib MAIC

Treatment Comparison	Comparator Study	How was matching on eligibility criteria performed?	Number of patients in ASCEMBL after matching^a^
Asciminib vs Ponatinib	PACE	ASCEMBL allowed patients with CCyR at baseline whereas PACE excluded patients with CCyR. 19 patients from ASCEMBL had CCyR at baseline; 35 patients had unknown CCyR status. All patients having confirmed CCyR at baseline or unknown CCyR status (n = 54) were removed from ASCEMBL. The following scenarios were considered in addition to the base case: Removal of patients having confirmed CCyR at baseline (*n* = 19)	103
Asciminib vs Nilotinib	Giles et al. (2010)	ASCEMBL and Giles et al. (2010) had similar eligibility criteria. Thus, no patients were removed from ASCEMBL. A scenario where patients achieving major cytogenic response were removed was considered separately.^b^	157
Asciminib vs Dasatinib	Rossi et al. (2013) Tan et al. (2019)	ASCEMBL and both studies had similar eligibility criteria. Thus, no patients were removed from ASCEMBL.	157
Asciminib vs Nilotinib/Dasatinib	Ibrahim et al. (2010)	ASCEMBL and Ibrahim et al. (2010) had similar eligibility criteria. Only CCyR response data were available from Ibrahim et al. (2010). CCyR response data were only available for the CCyR subgroup in ASCEMBL. Thus, the ASCEMBL CCyR subgroup (n = 103) was considered for the MAIC.	103

^a^There were 157 patients in ASCEMBL prior to the matching process

^b^21% of patients who were on nilotinib (in the study conducted by Giles et al. 2010) were able to achieve major cytogenic response. However, it was not known if the response achieved in these patients was the best response achieved or was achieved at the start of nilotinib therapy. Thus, major cytogenic response was assumed to be at the start of the nilotinib treatment for the base case analysis as a conservative assumption

*CCyR* complete cytogenic response; *MAIC* matching-adjusted indirect comparison; *TTD* time to treatment discontinuation

Prior to conducting the analyses, potentially important prognostic factors, which were identified based on an SLR for clinical outcomes in CP-CML patients who had received ≥ 2 TKIs prior to study entry and by consulting clinical experts, were ranked in order of importance based on their expected impact on outcome. Nine factors were identified to be the most important prognostic variables: gender, baseline age, race, partial cytogenetic response (PCyR) at baseline, number of prior TKIs received before study entry, resistance to prior TKIs, intolerance to prior TKIs, mutation status, and Eastern Cooperative Oncology Group Performance Status (ECOG PS). From the various MAIC simulations executed for each comparison, the most suitable scenario was selected based on convergence of the model with maximum number of matching baseline variables and an optimal ESS.

Relative efficacy for asciminib versus comparator TKIs was determined for the binary endpoints MMR and CCyR rates by estimating the relative risks (RRs) and their 95% confidence intervals (CIs). The difference between the compared groups was considered statistically significant when the 95% CI range did not include “1.” Of note, cumulative incidence curves to MMR and CCyR were only available for asciminib and ponatinib. The curves reported for ponatinib were digitized using WebPlotDigitizer (v4.5) to retrieve relevant data points required to calculate RR. RRs comparing asciminib versus the remaining comparators were calculated as a ratio of risks using comparator response data reported at specified timepoints.

Relative efficacy is typically determined for TTD (i.e., a time-to-event outcome) by estimating a hazard ratio (HR) using a Cox proportional hazards model. This method requires that Kaplan–Meier (KM) curves are reported for the competing treatments, so that they can be leveraged to capture the data at every timepoint. Although a TTD KM curve was reported in ASCEMBL, none of the comparator trials reported a TTD KM curve. Thus, it was not possible to calculate an indirect treatment effect estimate using a Cox proportional hazards model. Alternatively, the median TTD of the adjusted population receiving asciminib was compared to the median treatment duration reported for each of the comparators.

All analyses were conducted using R (R Core Team, Vienna, Austria: http://www.R-project.org/) based on the methods developed by (Signorovitch et al. 2010, 2012), with an adapted sample code from the NICE Decision Support Unit (DSU) Technical Support Document 18 for calculating MAIC weights (Phillippo et al. 2016, 2019).

Quantitative approaches to assess the numerical feasibility of conducting a MAIC (Glimm and Yau 2022) were recently introduced and validated in R code. By the means of numerical examination of the patient baseline characteristics, these approaches assessed whether sufficient overlap in patient characteristics between IPD and AD was present for a valid MAIC to proceed. It was necessary to perform these additional statistical tests of the data after clinical aspects of the comparability of the studies have been confirmed. This is because although the eligibility criteria of the different data sources can be similar, differences in the average patient characteristics may be present due to shifts in regions or centers. For the analysis, these statistical tests were conducted using the ASCEMBL IPD and the AD of each of the comparators and they confirmed that it was appropriate to conduct the MAIC.

## Results

### Study identification

The SLR identified five unique studies for the respective comparators of asciminib: ponatinib (PACE), nilotinib (Giles et al. 2010; Ibrahim et al. 2010), and dasatinib (Ibrahim et al. 2010; Rossi et al. 2013; Tan et al. 2019) (Table 1).

### Study and patient characteristics

An overview of the study and patient characteristics of the included studies is presented in Table 1. ASCEMBL was an open-label, phase III RCT, while all other comparator studies were single-arm and non-randomized. Namely, PACE and the study conducted by Giles et al. (2010) were phase II single-arm studies; the study conducted by Tan et al. (2019) was a retrospective study; and studies conducted by Rossi et al. (2013) and Ibrahim et. al., 2010 were prospective studies. There was variation in follow-up and treatment durations across the included studies. There was also variation in the requirement for prior treatment among the included studies. ASCEMBL recruited patients who had ≥ 2 prior TKIs at study entry, with failure or intolerance to the last line of TKI. However, patients in PACE were required either to have disease that was resistant/intolerant to dasatinib or nilotinib or to have developed the T315I mutation after any TKI therapy. Moreover, Giles et al. (2010) recruited patients whose disease was resistant/intolerant to imatinib and had failed to respond to dasatinib. Tan et al. (2019) recruited patients whose disease had failed either imatinib alone or both imatinib and nilotinib. Although Rossi 2013 and Ibrahim 2010 recruited patients who received 2 prior lines of TKI, these patients had to have disease that failed first-line imatinib and then failed either nilotinib or dasatinib during second line of treatment. Three studies (including ASCEMBL) excluded patients harboring the T3151 mutation. ASCEMBL allowed patients with CCyR at baseline, whereas PACE excluded them; the remaining studies did not report criteria pertaining to these patients. ASCEMBL reported a median age of 50 years, which was similar to a median age of 50 years reported by Tan et al. (2019); the remaining comparator studies reported a higher median age at baseline. ASCEMBL and PACE both required patients to have an ECOG PS score of 0–2; the remaining studies did not specify this criterion.

### Outcome availability and definitions

Only cumulative response outcomes reported by 6 and 12 months were included in the analysis. AD for comparison of response were only available from the PACE (Cohort A; patients receiving ≥ 3L treatment) (ponatinib), Ibrahim et al. (2010) (pooled nilotinib/dasatinib), and Tan et al. (2019) (dasatinib) studies. A comparison of treatment response was conducted using the available AD for CCyR and MMR rates by 6 and 12 months from PACE (cohort A) (ponatinib), CCyR and MMR rates by 6 months from Tan et al. (2019) (dasatinib), and CCyR by 6 and 12 months from Ibrahim et al. (2010) (pooled nilotinib/dasatinib). In addition to these comparisons, an analysis comparing asciminib and ponatinib was conducted, where 13 ponatinib-pretreated patients were further excluded from ASCEMBL. This was done to better align patients in ASCEMBL to the eligibility criteria of PACE, which excluded ponatinib-pretreated patients. AD for comparison of TTD were only available from the PACE (Cohort A + B; patients either receiving ≥ 3L treatment or harboring the T315I mutation) (ponatinib), Giles et al. (2010) (nilotinib), and Rossi et al. (2013) (dasatinib) studies.

Definitions of MMR were similar across the studies reporting this outcome. ASCEMBL, PACE, and Tan et al. (2019) classified MMR according to BCR::ABL1 transcript levels according to the International Scale (IS). Definitions of CCyR were mostly similar across the studies reporting this outcome. ASCEMBL and PACE both defined CCyR as the absence of Ph + cells in at least 20 metaphases. However, Ibrahim et al. (2010) defined CCyR as the absence of Ph + cells in two consecutive bone marrow examinations with at least 30 metaphases. Tan et al. (2019) was unclear in defining CCyR. Definitions of TTD were mostly similar across the studies that reported this outcome, with all definitions specifying treatment failure and unacceptable toxicity as reasons for discontinuation. ASCEMBL was the only study to define treatment failure according to the ELN recommendations. However, ASCEMBL and PACE reported similar definitions of TTD, specifying additional reasons for discontinuation such as: death, disease progression, loss to follow-up, withdrawal of consent, and discretion of the investigator. Among the included studies, ASCEMBL was the only study to report a TTD curve. Thus, it was not possible to calculate an indirect treatment effect estimate using a Cox proportional hazards model. Alternatively, the median TTD of the adjusted population receiving asciminib was compared to the median treatment duration reported for each of the comparators.

### Matching-adjusted indirect comparisons

#### Asciminib vs ponatinib (MMR and CCyR)

In the adjusted base case analysis, 54 patients in ASCEMBL who did not satisfy PACE’s inclusion criteria for CCyR were excluded (Table 2). Further exclusions were not required, as all other key eligibility criteria were similar between the trials. After reweighting the remaining 103 patients from ASCEMBL to align with the population of interest in PACE (Cohort A), all the baseline characteristics of interest were similar (Table 3), accompanied by a 48% reduction in ESS for the asciminib population. The following factors were included in the base case analysis based on convergence of the model, including the maximum number of variables, and obtaining an optimal ESS: baseline age, ECOG PS, proportion of patients with no mutations, PCyR rate, and proportion of patients with two prior TKIs (Table 3). The overall ranges from various scenarios are presented in Online Resource 5.

**Table 3:** Comparison of baseline characteristics before and after MAIC

Trial/Study		Race (White)	Male	Median age	ECOG = 0	No mutation	PCyR	MCyR	Resistance		Intolerance		Prior TKIs
									Nilotinib/dasatinib		Nilotinib/dasatinib		TKIs = 2
*Comparison: asciminib vs ponatinib (cohort A + B*)													
PACE	*N* = 270	80.9%	53.3%	60	70.0%	51.1%	19.6%	–	79.6%	–	14.4%	-	33.7%
ASCEMBL – pre-MAIC	*N* = 103	71.8%	43.7%	53	76.7%	86.4%	24.3%	–	69.9%	–	39.8%	–	53.4%
ASCEMBL – post-MAIC	ESS = 31	76.5%	34.6%	60	70.0%	51.1%	19.6%	–	79.6%	–	14.4%	–	33.7%
*Comparison: asciminib vs ponatinib (cohort A)*													
PACE	*N* = 203	85.7%	46.8%	61	68.5%	67.0%	19.2%	–	–	–	–	–	31.5%
ASCEMBL – pre-MAIC	*N* = 103	71.8%	43.7%	53	76.7%	86.4%	24.3%	–	–	–	–	–	53.4%
ASCEMBL – post-MAIC	ESS = 53	73.9	42.3	61	68.5%	67.0%	19.2%	–	–	–	–	–	31.5
Comparison: asciminib vs nilotinib									Imatinib	Dasatinib	Imatinib	Dasatinib	TKIs = 2
Giles et al. 2010	*N* = 39	–	–	62	64%	33%	–	21%	85%	31%	15%	67%	100%
ASCEMBL – pre-MAIC	*N* = 157	–	–	52	80%	87%	–	28%	54%	45%	54%	35%	52%
ASCEMBL – post-MAIC	ESS = 48	–	–	53	77%	95%	–	21%	32%	31%	74%	67%	100%
*Comparison: asciminib vs dasatinib*									Imatinib	Nilotinib	Imatinib	Nilotinib	TKIs = 2
Rossi et al. 2013	*N* = 34	–	27%	60	–	50%	–	–	94%	59%	6%	50%	100%
ASCEMBL – pre-MAIC	*N* = 157	–	52%	52	–	87%	–	–	54%	38%	54%	23%	43%
ASCEMBL – post-MAIC	ESS = 61	−	53%	46	–	79%	–	–	42%	59%	39%	12%	100%
*Comparison: asciminib vs dasatinib*													
Tan et al. 2019	*N* = 24	–	63%	50	–	54%	–	–	–	59%	–	50%	100%
ASCEMBL – pre-MAIC	*N* = 157	−	52%	52	–	87%	–	–	–	38%	–	23%	43%
ASCEMBL – post-MAIC	ESS = 23	−	63%	50	–	54%	–	–	–	59%	–	50%	100%
*Comparison: asciminib vs nilotinib/dasatinib*									Nilotinib/dasatinib		Nilotinib/dasatinib		TKIs = 2
Ibrahim et al. 2010	*N* = 26	–	54%	64	–	54%	–	–	27%	–	65%	–	100%
ASCEMBL – pre-MAIC	*N* = 103	–	44%	53	–	86%	–	–	70%	–	40%	–	53%
ASCEMBL – post-MAIC	ESS = 35	–	44%	51	–	92%	–	–	27%	–	65%	–	100%

*ECOG* Eastern Cooperative Oncology Group; *ESS* effective sample size; *MAIC* matching-adjusted indirect comparison; *MCyR* major cytogenetic response; *PCyR* partial cytogenetic response; *TKI* tyrosine kinase inhibitor

For both the unadjusted and adjusted comparisons, the observed MMR and CCyR rates, and the relative treatment effect estimates (RRs) are summarized in Table 4.

**Table 4:** Overview of relative efficacy of different interventions comparing the rate of MMR/CCyR by 6 months and 12 months

Study	MMR				CCyR			
	6 months		12 months		6 months		12 months	
	% patients	RR [95% CI]	% patients	RR [95% CI]	% patients	RR [95% CI]	% patients	RR [95% CI]
Asciminib vs ponatinib (cohort A)*								
ASCEMBL – pre-MAIC	28%	-	35%	-	41%	–	46%	–
ASCEMBL – post-MAIC	29%	1.55 [1.02, 2.36]	34%	1.48 [1.03, 2.14]	38%	1.11 [0.81, 1.52]	42%	0.97 [0.73, 1.28]
PACE	19%		23%		34%		43%	
Asciminib vs nilotinib/dasatinib								
ASCEMBL – pre-MAIC	–	–	–	–	41%	–	46%	–
ASCEMBL – post-MAIC	–	–	–	–	54%	3.57 [1.42, 8.98]	63%	2.03 [1.12, 3.67]
Ibrahim et al. (2010)	–	–	–	–	15%		31%	
Asciminib vs dasatinib								
ASCEMBL – pre-MAIC	27%	–	–	–	–	–	–	–
ASCEMBL – post-MAIC	27%	1.29 [0.57, 2.93]	–	–	–	–	–	–
Tan et al. (2019)	21%		–	–	–	–	–	–
Rossi et al. (2013)	–	–	–	–	–	–	–	–

^*^PACE cohort A (*N* = 203) includes patients on ≥ 3L CP-CML therapy

*CCyR* complete cytogenetic response; *CI* confidence interval; *MAIC* matching-adjusted indirect comparison; *MCyR* major cytogenetic response; *RR* risk ratio; *TKI* tyrosine kinase inhibitor; *TTD* time to treatment discontinuation

By 6 months, the unadjusted MMR and CCyR rates among patients receiving asciminib were 28% and 41%, respectively. In comparison, 19% and 34% of patients treated with ponatinib (Cohort A) achieved MMR and CCyR, respectively. After base case adjustment, patients treated with asciminib demonstrated significant improvement over ponatinib (Cohort A) in terms of MMR by 6 months (RR: 1.55, 95% CI: 1.02, 2.36). By the same timepoint, asciminib showed numerical improvement over ponatinib (Cohort A) in terms of CCyR (RR: 1.11, 95% CI: 0.81, 1.52).

**Fig. 1:**
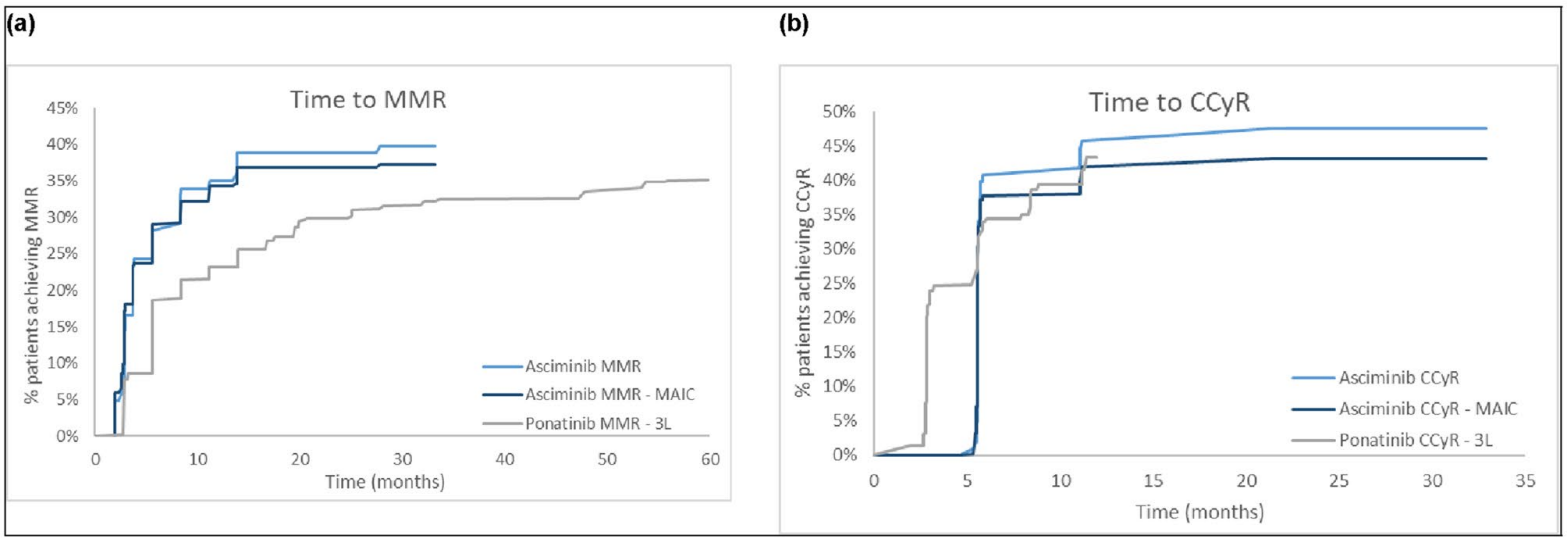
Cumulative response curves for time to MMR and CCyR curves. **a** MMR, asciminib (ASCEMBL) vs ponatinib (PACE cohort A). **b** CCyR, asciminib (ASCEMBL) vs ponatinib (PACE cohort A). PACE cohort A (*N* = 203) includes patients on ≥ 3L CP-CML therapy. *3L* third line; *CCyR* complete cytogenetic response; *MAIC* matching-adjusted indirect comparison; *MMR* major molecular response

By 12 months, the unadjusted MMR and CCyR rates among patients receiving asciminib were 35% and 46%, respectively. In comparison, 23% and 43% of patients treated with ponatinib (Cohort A) achieved MMR and CCyR, respectively. After base case adjustment, asciminib demonstrated significant improvement over ponatinib (Cohort A) in terms of MMR by 12 months (RR: 1.48, 95% CI: 1.03, 2.14). However, ponatinib demonstrated slight improvement over asciminib in terms of CCyR by 12 months although this result was not statistically significant (RR: 0.97, 95% CI: 0.73, 1.28). Moreover, asciminib had relatively favorable results compared with ponatinib (Cohort A) for the times taken to achieve MMR by 6 and 12 months, and CCyR by 6 months (Fig. 1).

An additional analysis further excluding 13 ponatinib-pretreated patients in ASCEMBL was conducted. After reweighting the remaining 90 patients from ASCEMBL to align with the population of interest in PACE (Cohort A), all the baseline characteristics of interest were similar (Table 3), accompanied by a 58% reduction in ESS for the asciminib population. Asciminib demonstrated significant improvements over ponatinib (Cohort A) in terms of MMR by both 6 (RR: 1.68, 95% CI: 1.1, 2.55) and 12 months (RR: 1.72, 95% CI: 1.2, 2.45). Asciminib demonstrated numerical improvements over ponatinib (Cohort A) in CCyR by both 6 (RR: 1.15, 95% CI: 0.84, 1.59) and 12 months (RR: 1.03, 95% CI: 0.78, 1.36). Detailed results of the additional analysis are presented in Online Resource 6 and Online Resource 7

### Asciminib vs ponatinib (TTD)

Similar to what was observed for matching according to PACE (Cohort A), 54 patients in ASCEMBL who did not satisfy PACE’s inclusion criteria for CCyR were excluded from the ASCEMBL in the base case analysis (Table 2). Further exclusions were not required, as all other key eligibility criteria were similar between the trials. After reweighting the remaining 103 patients from ASCEMBL to align with the population of interest in PACE (Cohort A + B), all the baseline characteristics of interest were similar (Table 3), accompanied by a 70% reduction in ESS for the asciminib population. The following factors were included in the base case analysis based on convergence of the model, including the maximum number of variables, and obtaining an optimal ESS: baseline age, ECOG status, proportion of patients with no mutations, PCyR rate, resistance or intolerance to prior nilotinib or dasatinib, and proportion of patients with two prior TKIs (Table 3).

**Fig. 2:**
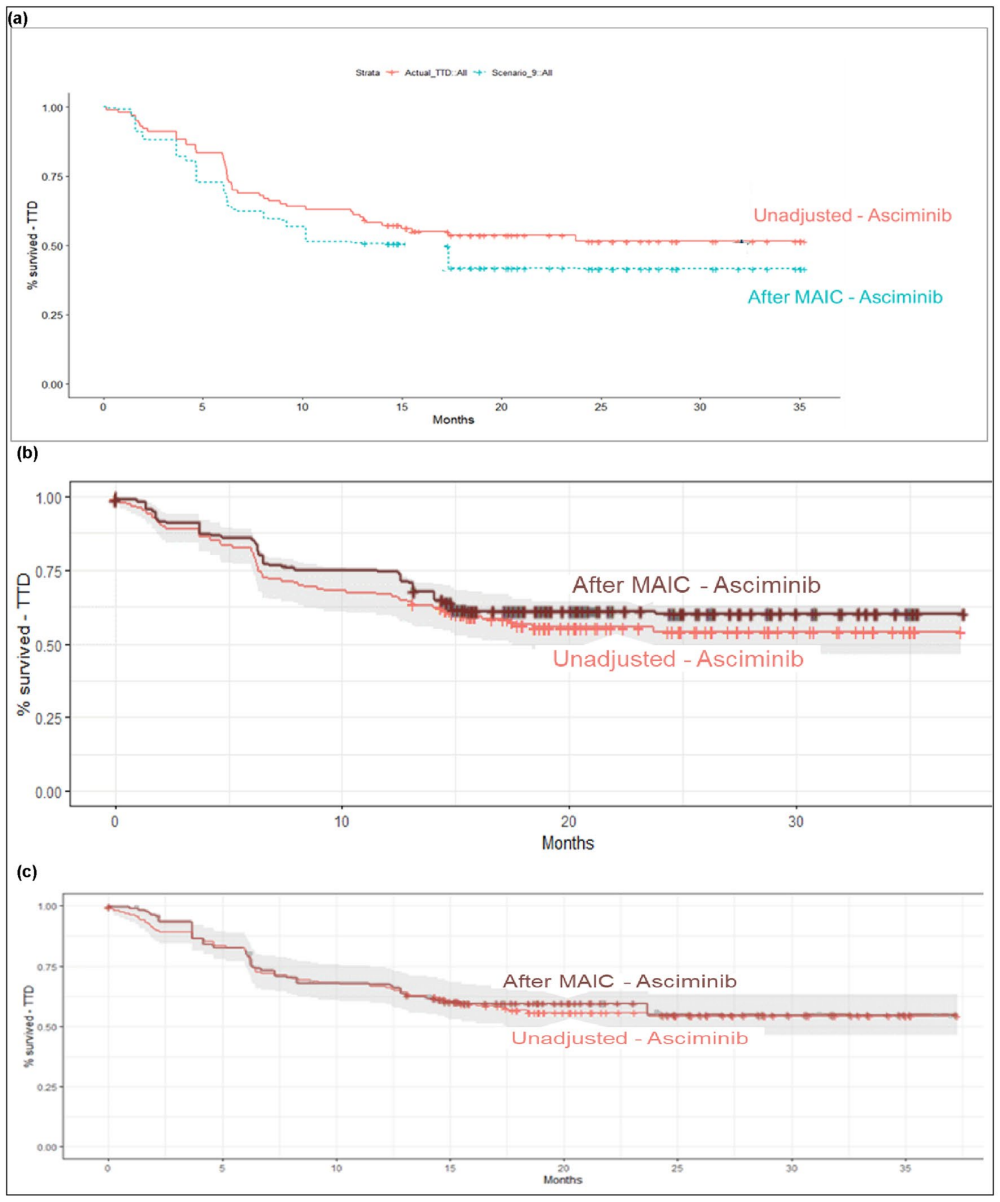
TTD KM curves before and after MAIC. **a** Asciminib (ASCEMBL) vs Ponatinib (PACE cohort A + B). **b** Asciminib (ASCEMBL) vs Nilotinib (Giles et al. 2010). **c** Asciminib (ASCEMBL) vs Dasatinib (Rossi et al. 2013). *KM* Kaplan–Meier; *MAIC* matching-adjusted indirect comparison; *TTD* time to treatment discontinuation

Prior to base case adjustment, the median TTD for patients receiving asciminib was not reached in the ASCEMBL trial. A median TTD of 32.1 months (published as ‘median treatment duration’) was reported for patients receiving ponatinib in the PACE (Cohort A + B) trial. After adjusting the ASCEMBL population to align with that of PACE (Cohort A + B), the median TTD for asciminib was 15.5 months. After the base case adjustment, the TTD curve for asciminib shifted downwards; this implied that the adjustment resulted in an overall shorter TTD among patients receiving asciminib matched to PACE (Cohort A + B) compared to the ASCEMBL population prior to adjustment. Figure 2 presents the KM curve comparing the TTD for asciminib (ESS = 31) with that of ponatinib (PACE [Cohort A + B]; *N* = 270).

### Asciminib vs nilotinib (TTD)

In the adjusted base case analysis, ASCEMBL and Giles et al. (2010) (nilotinib) had similar eligibility criteria; therefore, exclusions from the ASCEMBL IPD were not required (Table 2). After reweighting the 157 patients from ASCEMBL to align with the population of interest in Giles et al. (2010), all the baseline characteristics of interest were similar (Table 3), accompanied by a 69% reduction in ESS for the asciminib population. The following factors were included in the base case analysis based on convergence of the model, including the maximum number of variables, and obtaining an optimal ESS: baseline major cytogenetic response rate (MCyR), resistance or intolerance to dasatinib, and proportion of patients with two prior TKIs (Table 3). The overall ranges from various scenarios are presented in Online Resource 5.

Prior to base case adjustment, the median TTD for patients receiving asciminib in ASCEMBL was not reached. The median TTD (originally reported as median treatment duration) was reported as 11 months for patients receiving nilotinib in Giles et al (2010). After adjusting the ASCEMBL population to align with the Giles et al. (2010), the median TTD for asciminib remained unreached. After this adjustment, the TTD curve for asciminib shifted upward; this implied that the adjustment resulted in an overall longer TTD among patients receiving asciminib compared to the ASCEMBL population prior to adjustment. Figure 2 presents the KM curve comparing the TTD for asciminib (ESS = 48) with that of nilotinib (Giles et al. 2010; *N* = 39).

### Asciminib vs nilotinib/dasatinib (CCyR)

In the adjusted base case analysis, ASCEMBL and the study by Ibrahim et al. (2010) (pooled nilotinib/dasatinib) had similar eligibility criteria; therefore, exclusions from the ASCEMBL IPD were not required. However, only the CCyR outcome was available in Ibrahim et al. (2010) and given that CCyR data was only available for the CCyR subgroup in ASCEMBL, only this population was considered in the analysis (Table 2). After reweighting the 103 patients from ASCEMBL to align with the population of interest in Ibrahim et al. (2010), all the baseline characteristics of interest were similar (Table 3), accompanied by a 66% reduction in ESS for the asciminib population. The following factors were included in the base case analysis based on convergence of the model, including the maximum number of variables, and obtaining an optimal ESS: resistance or intolerance to prior nilotinib/dasatinib and the proportion of patients with two prior TKIs (Table 3). The overall ranges from various scenarios are presented in Online Resource 5.

For both the unadjusted and adjusted comparisons, the CCyR rates and the relative treatment effect estimates (RRs) are summarized in Table 4.

By 6 months and 12 months, the unadjusted CCyR rates among patients receiving asciminib were 41% and 46%, respectively. In comparison, 15% and 31% treated with nilotinib/dasatinib achieved CCyR by 6 months and 12 months, respectively. After base-case adjustment, patients treated with asciminib demonstrated significant improvements over nilotinib/dasatinib in terms of CCyR by 6 months (RR: 3.57, 95% CI: 1.42, 8.98) and 12 months (RR: 2.03, 95% CI: 1.12, 3.67) (Table 4).

### Asciminib vs dasatinib (MMR)

In the adjusted base case analysis, ASCEMBL and the study by Tan et al. (2019) had similar eligibility criteria; therefore, exclusions from the ASCEMBL IPD were not required (Table 2). After reweighting the 157 patients from ASCEMBL to align with the population of interest in Tan et al. 2019, all the baseline characteristics of interest were similar (Table 3), accompanied by an 85% reduction in ESS for the asciminib population. The following factors were included in the base case analysis based on convergence of the model, including the maximum number of variables, and obtaining an optimal ESS: baseline age, proportion of male patients, proportion of patients with no mutations, resistance or intolerance to nilotinib, and proportion of patients with two prior TKIs (Table 3). The overall ranges from various scenarios are presented in Online Resource 5.

For both the unadjusted and adjusted comparisons, MMR and the relative treatment effect estimates (RRs) are summarized in Table 4.

By 6 months, the unadjusted MMR rate among patients receiving asciminib was 27%. In comparison, 21% treated with dasatinib achieved MMR by 6 months. After base-case adjustment, patients treated with asciminib demonstrated numerical improvement over dasatinib in terms of MMR by 6 months (RR: 1.52, 95% CI: 0.66, 3.53), respectively.

### Asciminib vs dasatinib (TTD)

In the adjusted base case analysis, ASCEMBL and Rossi et al. (2013) (dasatinib) had similar eligibility criteria; therefore, exclusions from the ASCEMBL IPD were not required (Table 2). After reweighting the 157 patients from ASCEMBL to align with the population of interest in Rossi et al. (2013), all the baseline characteristics of interest were similar (Table 3), accompanied by a 61% reduction in ESS for the asciminib population. The following factors were included in the base case analysis based on convergence of the model, including the maximum number of variables, and obtaining an optimal ESS: resistance to nilotinib and proportion of patients with two prior TKIs (Table 3). The overall ranges from various scenarios are presented in Online Resource 5.

Prior to base case adjustment, the median TTD for patients receiving asciminib was not reached. The median TTD (originally reported as median treatment duration) was reported as 14 months for patients receiving dasatinib. After adjusting the ASCEMBL population to align with that of Rossi et al. (2013), the median TTD for asciminib remained unreached. After this adjustment, the TTD curve for asciminib did not shift substantially; this implied that the adjustment did not have an appreciable impact on TTD among patients receiving asciminib compared to the ASCEMBL population prior to adjustment. Figure 2 presents the KM curve comparing the TTD for asciminib (ESS = 61) with that of dasatinib (Rossi et al. (2013); *N* = 34).

### Naïve comparison of safety outcomes

Although safety outcomes were of interest for the analyses, there were several challenges in adjusting for the differences in baseline characteristics. Across the included studies, there were several differences in key factors influencing safety outcomes, such as: definition and classification of AEs, frequency and severity of AEs, and duration of treatment exposure. As a result, adjusting for differences in baseline characteristics was not feasible and MAICs could not be used to assess safety outcomes. Alternatively, unadjusted data on AEs were compared naively using the prescribing information of ASCEMBL and published comparator studies.

Among the included studies, asciminib had a better overall safety profile. In terms of treatment discontinuation, patients treated with asciminib demonstrated favorable outcomes when compared to dasatinib, ponatinib, and nilotinib.

Detailed results of the naïve safety analyses are presented in Online Resource 8.

## Discussion

With a novel mechanism of action, asciminib exhibited a favorable efficacy and safety profile in the phase 3 ASCEMBL trial. In addition to gaining FDA approval for the treatment of patients with CP-CML after ≥ 2 TKIs (Novartis 2021) who do not harbor the T315I mutation, it was recently recommended for reimbursement in the UK (NICE 2022) and received approval from the Committee for Medicinal Products for Human Use (CHMP) of the European Medicines Agency (EMA) (Novartis 2022). The approval and recommendations of asciminib represent a promising advancement in the ≥ 3L CP-CML treatment landscape. In the absence of head-to-head RCTs, the present analyses assessed the effectiveness of asciminib in those who do not harbor the T315I mutation versus alternative ≥ 3L TKIs in the treatment of CP-CML. Published AD from the comparator studies and IPD from ASCEMBL enabled the correction of cross-trial imbalances in patient characteristics through the conduct of unanchored MAICs. The results from these analyses demonstrated that asciminib had superior efficacy for most of the outcomes when compared with the conventionally used ≥ 3L TKIs for CP-CML. Moreover, a naïve comparison showed that asciminib had a favorable safety profile when compared to the other TKIs. However, these analyses are subject to a few limitations arising from their methodology and must be considered during the interpretation of their results. Albeit these limitations, the present analyses facilitated treatment comparisons between asciminib and key comparators based on currently available data, thus addressing the noted lack of published head-to-head RCTs evaluating TKIs in ≥ 3L CP-CML. The results of these analyses can be used to aid key stakeholders involved in healthcare decision-making related to more well-defined treatment pathways for patients with ≥ 3L CP-CML.

These analyses showed that asciminib mostly demonstrated favorable response outcomes when compared with key comparator treatments for ≥ 3L CP-CML. Significant improvements were especially noted in MMR rate for asciminib over ponatinib (Cohort A). Asciminib also demonstrated numerical improvements in CCyR rate by 6 months when compared to ponatinib (PACE [Cohort A]) and significant improvements by both 6 and 12 months when compared to pooled patients receiving either nilotinib or dasatinib. However, CCyR rate by 12 months for post-adjustment asciminib was slightly lower when compared to ponatinib (PACE [Cohort A]). Asciminib had relatively favorable results compared with ponatinib (PACE [Cohort A]) for the times taken to achieve MMR by 6 and 12 months, and CCyR by 6 months.

In an additional analysis comparing asciminib and ponatinib and excluding ponatinib-pretreated patients in ASCEMBL, the improvements of asciminib over ponatinib in achieving MMR and CCyR were further highlighted. Among the excluded patients, only one patient received asciminib as a ≥ 3L therapy, whereas the remaining 12 patients were highly pretreated (fourth- or later line [≥ 4L] therapy). The exclusion of these highly pretreated patients may have contributed to the improved relative efficacy of asciminib compared to ponatinib and presented a more objective comparison of the two treatments.

Where feasible, the results of the analyses showed that asciminib demonstrated improvements in MMR when compared to ponatinib, dasatinib, and nilotinib/dasatinib. These results are of importance as MMR is acknowledged as a well-established surrogate for long-term survival outcomes (NICE 2022).

Prior to adjustment, the median TTD for asciminib was not reached in the ASCEMBL trial with a follow-up of least 48 weeks. Adjustment of the ASCEMBL population according to the nilotinib and dasatinib study populations resulted in a median TTD that was not yet reached for asciminib. However, a shorter median TTD was noted for asciminib when compared to ponatinib. A longer TTD observed for ponatinib could be due to the reduction in the number of patients receiving asciminib after adjustment according to patients receiving ponatinib (PACE [Cohort A + B]); thus, patients with better outcomes in ASCEMBL may have been eliminated. In the PACE trial, a clear difference in response outcomes (both MMR and CCyR) was observed when comparing patients with and without T315I mutation. A better response observed with presence of the T315I mutation may encourage patients to remain on ponatinib, especially given heavy pre-treatment and the lack of alternative subsequent treatments other than allo-SCT (Boddu et al. 2018). Thus, the overall TTD results may be skewed in favor of ponatinib, when comparing the mixed group of patients in the PACE (Cohort A + B) trial with those of ASCEMBL (which excluded patients who harbored T315I mutation).

Due to the challenges of comparing the safety outcomes using MAICs, unadjusted data on AEs taken from the prescribing information and published studies were compared naively. Overall, asciminib demonstrated a favorable safety profile when compared to the other TKIs. Notably, patients on asciminib had lower rates of treatment discontinuation due to AEs. Favorable safety outcomes may be attributed to the novel mechanism of action of asciminib specific to ABL kinases and may provide an advantage over key comparators in ≥ 3L CP-CML. Ponatinib, a third-generation TKI therapy, is approved in patients with CP-CML resistant or intolerant to second-generation TKIs or in CML patients harboring the T315I mutation. However, safety concerns were raised due to potential life-threatening AEs, including arterial occlusive events, venous thromboembolic events, and heart failure; thus, the use of ponatinib was restricted in patients with prior cardiovascular risk factors (Hochhaus et al. 2020a, b; Cortes and Lang 2021). With a promising safety profile, asciminib may allow a broader patient population to benefit from its treatment without restriction from co-morbidities and tolerability challenges. However, the results of the naïve comparisons must be interpreted with caution as they are limited by the heterogeneity of study variables, definitions of AEs used, and limited data in the available publications.

A key strength of the current analysis was the incorporation of IPD from the ASCEMBL trial, which was adjusted according to key comparator populations to facilitate treatment comparisons involving asciminib. The logistic propensity score model used to estimate weights for the IPD from ASCEMBL for alignment with weighted mean baseline characteristics of the comparator population was consistent with the recommendations from the DSU commissioned by NICE (Phillippo et al. 2016, 2018). Despite a lack of published consensus on the appropriate reduction in ESS, these comparisons with asciminib generated ESS estimates which aligned with those found in previously published MAICs (Levy et al. 2019; Phillippo et al. 2019). The post-MAIC reductions in ESS, ranging between ~ 48% (asciminib vs ponatinib [PACE]) and ~ 85% (asciminib vs dasatinib [Tan et al. 2019]), aligned with reductions reported by Phillippo et al. 2019 (median: 74.2%; range: 7.9–94.1%) and Levy et al. 2019 (27.8%) (Levy et al. 2019; Phillippo et al. 2019).

Although this analysis was able to facilitate comparisons between asciminib and other available treatments for patients with ≥ 3L CP-CML, there are a few limitations that must be considered. Given that the analysis was limited to published AD for the comparators, the populations were balanced based on aggregate statistics rather than on the individual patients themselves. As a result, there may still be residual heterogeneity at the patient level contributing to potential bias in the treatment effect estimates. This limitation could be compensated for to some extent due to the robust approach used for selecting the prognostic factors and prioritizing them for the propensity model. However, it is unclear whether any unaccounted risk of bias was induced due to the differences in reporting of baseline characteristics and the definitions used in various studies. Moreover, although the present analysis aimed to adjust for all identified important patient characteristics relevant to the target population, the model did not converge when accounting for all these characteristics. Therefore, the present analysis aimed to balance the number of variables adjusted for while maintaining sufficient ESS.

Many studies did not have a sufficient overlap of patient population when compared with ASCEMBL; therefore, the ESS used in MAIC was smaller than the actual sample size of ASCEMBL. The smaller ESS sizes may have contributed to wider CIs, thus affecting the interpretation of the data. Although the adjustment of sample size accounted for the between-study differences and contributed to objective comparison, these results must be interpreted with caution. Despite this constraint, most of the important patient characteristics were retained during the present analysis. Thus, the analysis was still able to achieve balance between the number of variables adjusted for while maintaining a sufficient ESS aligning with those reported in previously published MAICs.

The MAIC was limited by the number of studies used to inform each treatment comparison, as most comparators were informed by single studies reporting on the comparator and meeting stringent criteria of the SLR and the analyses. Comparisons with nilotinib and dasatinib were informed by three retrospective studies having small sample sizes (range: 24–39 patients Giles et al. 2010; Ibrahim et al. 2010; Rossi et al. 2013)). Moreover, the present analysis could not include all studies reporting on mixed study populations containing ≥ 3L patients and patients not of interest, such as BYOND and OPTIC. This is because our criteria for MAICs required > 75% of the study population to be in ≥ 3L CP-CML, along with the availability of baseline characteristics for this target patient group to minimize heterogeneity (Online Resource 3). In addition, as none of the comparator studies reported KM curves for TTD, a time-to-event indirect comparative analysis could not be conducted with a Cox proportional hazards model and a HR was not calculated. Alternatively, the median TTD of post-adjustment asciminib was compared with the median treatment duration reported in each of the comparator studies. These comparisons may be limited by the differences in outcome definition, as the definition of median treatment duration may not necessarily equate to that of TTD; thus, results should be interpreted with caution. In addition to the discussed limitations, the analysis highlights the lack of RCTs evaluating treatments in the ≥ 3L CP-CML setting.

Despite the noted limitations, the present analyses facilitated treatment comparisons between asciminib and key comparators based on currently available data. The present analyses provide insights into the relative comparative effectiveness to address the paucity of head-to-head clinical trials evaluating asciminib against key comparators in patients with CP-CML in the third-line setting. The results of these analyses can be used to aid researchers, clinicians, and policymakers involved in healthcare decision-making. Such comparative analyses are vital in supporting the decisions of optimal treatment choice, which can help reduce the overall disease burden in patients who are resistant to multiple TKIs.

## Conclusion

The present analyses facilitated treatment comparisons between asciminib and key TKIs in ≥ 3L CP-CML, based on the most currently available data given a paucity of literature reporting ≥ 3L CP-CML treatments. These treatment comparisons showed that asciminib mostly demonstrated favorable outcomes in MMR rate, CCyR rate, and TTD, compared with other treatments used in patients with CP-CML previously treated with at least two TKIs. Of note, these analyses were limited by the ability to adjust for all characteristics, a lack of head-to-head trials, and limited studies to inform comparisons. The analysis aimed to include comparator populations where the majority of patients satisfied the inclusion criteria of ASCEMBL (treated with ≥ 2 TKIs and absence of T315I mutation). However, due to the consideration of mixed populations, some patients harboring the T315I mutation were included in the analysis. Despite these caveats, the comparative evidence from these MAIC analyses may help in bridging the gap to define a clear treatment pathway in patients whose disease fails ≥ 2 TKI therapies.

## Supplementary information

The online version contains the additional data and tables as supporting information.

## Supplementary Information

Below is the link to the electronic supplementary material.

Supplementary file1 (DOCX 194 KB)

## Data Availability

The data used to support the findings of this study are available upon reasonable request.

## References

[CR1] Acs (2018) Key Statistics for Chronic Myeloid Leukemia.American Cancer Society. <https://www.cancer.org/cancer/chronic-myeloid-leukemia/about/statistics.html> 20 Dec, 2021.

[CR2] Boddu P, Shah AR, Borthakur G, Verstovsek S, Garcia-Manero G, Daver N, Kadia T, Ravandi F, Jain N, Alhuraiji A, Burger J, Kornblau S, Pierce S, Dellasala S, Jabbour E, Kantarjian H, Cortes J (2018). Life after ponatinib failure: outcomes of chronic and accelerated phase CML patients who discontinued ponatinib in the salvage setting. Leuk Lymphoma.

[CR3] Bower H, Björkholm M, Dickman PW, Höglund M, Lambert PC, Andersson TML (2016). Life expectancy of patients with chronic myeloid leukemia approaches the life expectancy of the general population.

[CR4] Brümmendorf TH, Cortes JE, Milojkovic D, Gambacorti-Passerini C, Clark RE, Le Coutre PD, Garcia-Gutiérrez V, Chuah C, Kota V, Lipton JH (2020). Bosutinib (BOS) versus imatinib for newly diagnosed chronic phase (CP) chronic myeloid leukemia (CML): final 5-year results from the bfore trial. Blood.

[CR5] Cortes J, Lang F (2021). Third-line therapy for chronic myeloid leukemia: current status and future directions. J Hematol Oncol.

[CR6] Cortes JE, Saglio G, Kantarjian HM, Baccarani M, Mayer J, Boqué C, Shah NP, Chuah C, Casanova L, Bradley-Garelik B (2016). Final 5-year study results of DASISION: the dasatinib versus imatinib study in treatment-naïve chronic myeloid leukemia patients trial. J Clin Oncol.

[CR7] Dias S, Sutton AJ, Ades AE, Welton NJ (2013). Evidence synthesis for decision making 2: a generalized linear modeling framework for pairwise and network meta-analysis of randomized controlled trials. Med Decis Making.

[CR8] Garcia-Gutierrez V, Hernandez-Boluda JC (2019). Tyrosine kinase inhibitors available for chronic myeloid leukemia: efficacy and safety. Front Oncol.

[CR9] Garg RJ, Kantarjian H, O’brien S, Quintas-Cardama A, Faderl S, Estrov Z, Cortes J (2009). The use of nilotinib or dasatinib after failure to 2 prior tyrosine kinase inhibitors: long-term follow-up. Blood.

[CR10] Giles FJ, Abruzzese E, Rosti G, Kim DW, Bhatia R, Bosly A, Goldberg S, Kam GLS, Jagasia M, Mendrek W, Fischer T, Facon T, Dunzinger U, Marin D, Mueller MC, Shou Y, Gallagher NJ, Larson RA, Mahon FX, Baccarani M, Cortes J, Kantarjian HM (2010). Nilotinib is active in chronic and accelerated phase chronic myeloid leukemia following failure of imatinib and dasatinib therapy. Leukemia.

[CR11] Glimm E, Yau L (2022). Geometric approaches to assessing the numerical feasibility for conducting matching-adjusted indirect comparisons. Pharm Stat.

[CR12] Hehlmann R, Hochhaus A, Baccarani M, European L (2007). Chronic myeloid leukaemia. Lancet (london, England).

[CR13] Hochhaus A, La Rosee P, Muller MC, Ernst T, Cross NC (2011). Impact of BCR-ABL mutations on patients with chronic myeloid leukemia. Cell Cycle.

[CR14] Hochhaus A, Saglio G, Hughes TP, Larson RA, Kim DW, Issaragrisil S, Le Coutre PD, Etienne G, Dorlhiac-Llacer PE, Clark RE (2016). Long-term benefits and risks of frontline nilotinib vs imatinib for chronic myeloid leukemia in chronic phase: 5-year update of the randomized ENESTnd trial. Leukemia.

[CR15] Hochhaus A, Saussele S, Rosti G, Mahon FX, Janssen J, Hjorth-Hansen H, Richter J, Buske C, Committee EG (2017). Chronic myeloid leukaemia: ESMO clinical practice guidelines for diagnosis, treatment and follow-up. Ann Oncol.

[CR16] Hochhaus A, Baccarani M, Silver RT, Schiffer C, Apperley JF, Cervantes F, Clark RE, Cortes JE, Deininger MW, Guilhot F, Hjorth-Hansen H, Hughes TP, Janssen J, Kantarjian HM, Kim DW, Larson RA, Lipton JH, Mahon FX, Mayer J, Nicolini F, Niederwieser D, Pane F, Radich JP, Rea D, Richter J, Rosti G, Rousselot P, Saglio G, Saussele S, Soverini S, Steegmann JL, Turkina A, Zaritskey A, Hehlmann R (2020). European LeukemiaNet 2020 recommendations for treating chronic myeloid leukemia. Leukemia.

[CR17] Hochhaus A, Breccia M, Saglio G, Garcia-Gutierrez V, Rea D, Janssen J, Apperley J (2020). Expert opinion-management of chronic myeloid leukemia after resistance to second-generation tyrosine kinase inhibitors. Leukemia.

[CR18] Hoffmann VS, Lindoerfer D, Thaler J, Boris L, Melanthiou F, Mayer J, Everaus H, Guilhot J, Schubert-Fritschle G, Castagnetti F, Lejniece S, Griskevicius L, Thielen N, Sacha T, Hellmann A, Turkina AG, Zaritskey A, Bogdanovic A, Indrak K, Zupan I, Steegmann JL, Simonsson B, Clark R, Hoglund M, Hehlmann R, Hasford J, Baccarani M (2014). Incidence of CML in Europe – a comparison of 19 European countries with US SEER data. Blood.

[CR19] Ibrahim AR, Paliompeis C, Bua M, Milojkovic D, Szydlo R, Khorashad JS, Foroni L, Reid A, De Lavallade H, Rezvani K, Dazzi F, Apperley JF, Goldman JM, Marin D (2010). Efficacy of tyrosine kinase inhibitors (TKIs) as third-line therapy in patients with chronic myeloid leukemia in chronic phase who have failed 2 prior lines of TKI therapy. Blood.

[CR20] Ivanescu C, Skaltasa K, Kráľ P (2017). Acceptance of population-adjusted indirect treatment comparison methods in nice assessments. Value in Health.

[CR21] Jabbour E, Kantarjian H (2016). Chronic myeloid leukemia: 2016 update on diagnosis, therapy, and monitoring. Am J Hematol.

[CR22] Jabbour E, Kantarjian H (2020). Chronic myeloid leukemia: 2020 update on diagnosis, therapy and monitoring. Am J Hematol.

[CR23] Kantarjian HM, Deisseroth A, Kurzrock R, Estrov Z, Talpaz M (1993). Chronic myelogenous leukemia: a concise update. Blood.

[CR24] Kantarjian HM, Hughes TP, Larson RA, Kim D-W, Issaragrisil S, Le Coutre P, Etienne G, Boquimpani C, Pasquini R, Clark RE (2021). Long-term outcomes with frontline nilotinib versus imatinib in newly diagnosed chronic myeloid leukemia in chronic phase: ENESTnd 10-year analysis. Leukemia.

[CR25] Levy MY, Mcgarry LJ, Huang H, Lustgarten S, Chiroli S, Iannazzo S (2019). Benefits and risks of ponatinib versus bosutinib following treatment failure of two prior tyrosine kinase inhibitors in patients with chronic phase chronic myeloid leukemia: a matching-adjusted indirect comparison. Curr Med Res Opin.

[CR26] Manley PW, Barys L, Cowan-Jacob SW (2020). The specificity of asciminib, a potential treatment for chronic myeloid leukemia, as a myristate-pocket binding ABL inhibitor and analysis of its interactions with mutant forms of BCR-ABL1 kinase. Leuk Res..

[CR27] Mauro MJ (2021). Lifelong TKI therapy: how to manage cardiovascular and other risks. Hematology Am Soc Hematol Educ Program.

[CR28] NCCN (2021) Clinical Practice Guidelines in Oncology - Chronic Myeloid Leukemia (Version 3.2021 - January 13, 2021).National Comprehensive Cancer Network. https://www.nccn.org/professionals/physician_gls/pdf/cml.pdf 20 Dec, 2021.

[CR29] Nice (2022) Asciminib for treating chronic myeloid leukaemia after 2 or more tyrosine kinase inhibitors [ID3813]. 40504958

[CR30] Novartis (2021) FDA approves Novartis Scemblix® (asciminib), with novel mechanism of action for the treatment of chronic myeloid leukemia.https://www.novartis.com/news/media-releases/fda-approves-novartis-scemblix-asciminib-novel-mechanism-action-treatment-chronic-myeloid-leukemia 20 Dec, 2021.

[CR31] Novartis (2022) Novartis receives positive CHMP opinion for Scemblix®, a novel treatment for adult patients with chronic myeloid leukemia.https://www.novartis.com/news/media-releases/novartis-receives-positive-chmp-opinion-scemblix-novel-treatment-adult-patients-chronic-myeloid-leukemia 12 Sept, 2022.

[CR32] Phillippo DM, Ades AE, Dias S, Palmer S, Abrams KR, Welton NJ (2018). Methods for population-adjusted indirect comparisons in health technology appraisal. Med Decis Making.

[CR33] Phillippo DM, Dias S, Elsada A, Ades AE, Welton NJ (2019). Population Adjustment Methods for Indirect Comparisons: A Review of National Institute for Health and Care Excellence Technology Appraisals. Int J Technol Assess Health Care.

[CR34] Phillippo DM, Ades AE, Dias S, Palmer S, Abrams KR & Welton NJ (2016) NICE DSU technical support document 18: methods for population-adjusted indirect comparisons in submissions to NICE. Report by the Decision Support Unit

[CR35] Rea D, Mauro MJ, Boquimpani C, Minami Y, Lomaia E, Voloshin S, Turkina A, Kim DW, Apperley JF, Abdo A, Fogliatto LM, Kim DDH, Le Coutre P, Saussele S, Annunziata M, Hughes TP, Chaudhri N, Sasaki K, Chee L, Garcia-Gutierrez V, Cortes JE, Aimone P, Allepuz A, Quenet S, Bedoucha V, Hochhaus A (2021). A phase 3, open-label, randomized study of asciminib, a STAMP inhibitor, vs bosutinib in CML after 2 or more prior TKIs. Blood.

[CR36] Ribeiro BF, Miranda EC, Albuquerque DM, Delamain MT, Oliveira-Duarte G, Almeida MH, Vergilio B, Silveira RA, Oliveira-Duarte V, Lorand-Metze I, De Souza CA, Pagnano KB (2015). Treatment with dasatinib or nilotinib in chronic myeloid leukemia patients who failed to respond to two previously administered tyrosine kinase inhibitors–a single center experience. Clinics (sao Paulo).

[CR37] Rohrbacher M, Hasford J (2018). Epidemiology and etiology of chronic myeloid leukemia. Neoplastic diseases of the blood.

[CR38] Rossi AR, Breccia M, Abruzzese E, Castagnetti F, Luciano L, Gozzini A, Annunziata M, Martino B, Stagno F, Cavazzini F, Tiribelli M, Visani G, Pregno P, Musto P, Fava C, Sgherza N, Albano F, Rosti G, Alimena G, Specchia G (2013). Outcome of 82 chronic myeloid leukemia patients treated with nilotinib or dasatinib after failure of two prior tyrosine kinase inhibitors. Haematologica.

[CR39] Sasaki K, Strom SS, O’brien S, Jabbour E, Ravandi F, Konopleva M, Borthakur G, Pemmaraju N, Daver N, Jain P, Pierce S, Kantarjian H, Cortes JE (2015). Relative survival in patients with chronic-phase chronic myeloid leukaemia in the tyrosine-kinase inhibitor era: analysis of patient data from six prospective clinical trials. Lancet Haematol.

[CR40] Seer (2021) Cancer Stat Facts: Leukemia-Chronic Myeloid Leukemia (CML) [Internet]. National Cancer Institute Surveillance Epidemiology, and End Results Program.SEER. https://seer.cancer.gov/statfacts/html/cmyl.html. 20 Dec, 2021.

[CR41] Signorovitch JE, Wu EQ, Yu AP, Gerrits CM, Kantor E, Bao YJ, Gupta SR, Mulani PM (2010). Comparative effectiveness without head-to-head trials a method for matching-adjusted indirect comparisons applied to psoriasis treatment with adalimumab or etanercept. Pharmacoeconomics.

[CR42] Signorovitch JE, Sikirica V, Erder MH, Xie J, Lu M, Hodgkins PS, Betts KA, Wu EQ (2012). Matching-adjusted indirect comparisons: a new tool for timely comparative effectiveness research. Value Health.

[CR43] Tan J, Xue M, Pan J, Cen J, Qi X, Liu P, Zhao X, Wu P, Wang Q, Liu D, Liu Y, Chen S, Wang Z (2019). Responses to dasatinib as a second- and third-line tyrosine kinase inhibitor in chronic phase chronic myeloid leukaemia patients. Acta Haematol.

[CR44] Wylie AA, Schoepfer J, Jahnke W, Cowan-Jacob SW, Loo A, Furet P, Marzinzik AL, Pelle X, Donovan J, Zhu W, Buonamici S, Hassan AQ, Lombardo F, Iyer V, Palmer M, Berellini G, Dodd S, Thohan S, Bitter H, Branford S, Ross DM, Hughes TP, Petruzzelli L, Vanasse KG, Warmuth M, Hofmann F, Keen NJ, Sellers WR (2017). The allosteric inhibitor ABL001 enables dual targeting of BCR-ABL1. Nature.

