# Supplementary material for: Matching-adjusted indirect comparison of asciminib versus other treatments in chronic-phase chronic myeloid leukemia after failure of two prior tyrosine kinase inhibitors
Source: J Cancer Res Clin Oncol. 2023 Jan 28;149(9):6247–62. doi: 10.1007/s00432-022-04562-5 (PMC10356870; doi:10.1007/s00432-022-04562-5)
Supplement: Supplementary file 1 — Supplementary file1 (DOCX 194 KB) [file 432_2022_4562_MOESM1_ESM.docx]

## SUPPLEMENTARY DATA

**Matching-adjusted indirect comparison of asciminib versus other treatments in chronic-phase chronic myeloid leukemia after failure of two prior tyrosine kinase inhibitors**

**Journal:**

[Journal of Cancer Research and Clinical Oncology](https://www.springer.com/journal/432)

**Authors:**

Ehab Atallah^1^, Michael J. Mauro^2^, Andreas Hochhaus^3^, Carla Boquimpani^4,5^, Yosuke Minami^6^, Vikalp Kumar Maheshwari^7^, Lovneet Saini^7^, Regina Corbin^8^, Delphine Réa^9^

**Affiliations:**

^1^Medical College of Wisconsin, Milwaukee, WI, USA

^2^Memorial Sloan-Kettering Cancer Center, New York, NY, USA

^3^Universitätsklinikum Jena, Jena, Germany

^4^HEMORIO, State Institute of Hematology Arthur de Siquiera Cavalcanti, Rio de Janeiro, Brazil

^5^Oncoclínica Centro de Tratamento Oncológico, Rio de Janeiro, Brazil

^6^National Cancer Center Hospital East, Kashiwa, Japan

^7^Novartis Healthcare Pvt. Ltd, Hyderabad, India

^8^Novartis Services Inc., East Hanover, NJ, USA

^9^Adult Hematology Hôpital Saint-Louis and FiLMC, Paris, France

**Corresponding author:**

Dr. Ehab Atallah

Medical College of Wisconsin, Milwaukee, WI, USA

E-mail: eatallah@mcw.edu

#### Online Resource 1: PRISMA flow diagram

Online Resource Figure 1: PRISMA flow diagram for selection of studies identified from the searches conducted in May 2021


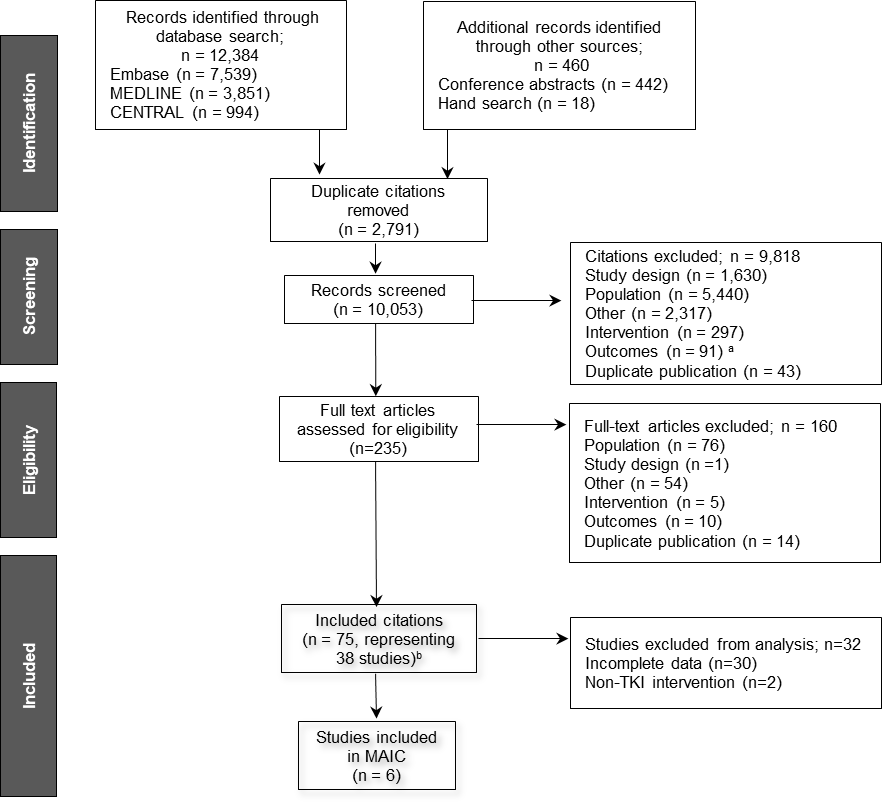


**Notes**: Exclusion criteria at first and second level of screening, Study design not of interest: Editorials, Comments, Letters, Surveys, Case studies; Population not of interest: Patients with diseases other than CML, pediatric patients, and CML in advanced phases; Intervention not of interest: treatments other than asciminib, TKIs, allo-SCT, HQP1351,omacetaxine, PF-114, hydroxycarbamide; Outcomes not of interest: outcomes other than MMR, MR4, CCyR, CHR, OS, PFS, EFS, TTD, AEs (any grade or grade 3-4), treatment discontinuation due to AEs; Other: Reviews, conference proceedings before 2017, and conference abstracts* a) Only conference proceedings were considered for exclusion by outcome at this stage; b) Including 11 full-text publications, 17 conference abstracts
* Proceedings were searched for select conferences, separately.

AE, adverse event, CCyR, complete cytogenetic response; CHR, complete hematologic response; EFS, event free survival; MAIC, matching-adjusted indirect comparisons; MCyR, major and complete cytogenetic response; MR, molecular response; OS, overall survival; PFS, progression free survival; TKIs, tyrosine kinase inhibitors; TTD, time to treatment discontinuation

#### Online Resource 2: Eligibility criteria

Online Resource Table 1: Eligibility criteria for inclusion of studies for matching-adjusted indirect comparison

| Criteria | Description | |
| --- | --- | --- |
| Population | *Inclusion criteria:*  Studies reporting outcomes for adult (≥18 years) CP-CML patients where ≥75% of patients had prior experience with ≥2 TKIs and did not harbor the T315I mutation  *Exclusion criteria:*  Studies with mixed population where <75% of patients matched the target population AND patient characteristics were not reported for target population^a^ | |
| Interventions | - Ponatinib - Dasatinib - Nilotinib | - Imatinib - Homoharringtonine (omacetaxine)* - Hydroxycarbamide |
| Comparators | - Placebo or best supportive care - Any intervention of interest | |
| Outcomes | Time to treatment discontinuation  CCyR and MMR by 6-months and 12-months  Safety outcomes (descriptive) | |
| Study design | *Inclusion criteria:*   - Interventional studies (randomized or non-randomized) - Observational studies (prospective or retrospective) - Cross-sectional studies   *Exclusion criteria:*   - Phase I trials - Dose-ranging, dose-finding, and dose-escalating trials | |
| Language | Only studies published in English will be included | |
| Time | No time restriction | |

*****Studies were identified, but not considered for current analysis
CCyR, complete cytogenetic response; CP-CML, chronic phase chronic myeloid leukemia; MMR, major molecular response; TKI, tyrosine kinase inhibitor.

#### Online Resource 3: List of studies excluded from matching-adjusted indirect comparison

Online Resource Table 2: Studies excluded from the analysis and their reasons for exclusion

| **List of excluded studies from the analysis (n=31)** | **Reason for exclusion** |
| --- | --- |
| - Khoury 2012 - Gambacorti-Passerini 2019 (BYOND study) - Takahashi 2017 - Garcia-Gutierrez 2019 - Tiribelli 2018 - Cortes 2019 - Latagliata 2021 - Luna 2020 - Jiang 2020 (CC201 study) | - Comparator or patient population not of interest (n=9) |
| - Garg 2009 - Ribeiro 2015 - Lee 2014 - Cortes 2012 (CML-202) - Iurlo 2019 (OITI) - Chan 2020 - Devos 2021 - Gugliotta 2020 - Chitanava 2020 - Guerci-Bresler 2021 (TOPASE study) | - These studies (n=10) recruited patients where <75% of the study population matched the target population for analysis and did not report baseline characteristics for the target population, separately |
| - Ongoren 2017 - Tojo 2017 | - These studies (n=2) were excluded because they did not report outcomes for >20 participants in the target population (i.e. less sample size) |
| - Swaminathan 2018 - Khan 2017 - Garcia-Gutierrez 2012 | - These studies (n=3) were excluded because they did not report patient characteristics for interventions of interest |
| - Deininger 2019 (X2101 study) - Cortes 2012 - Jiang 2019 - Turkina 2018 | - These studies (n=4) were excluded as they were phase I trials |
| - Cortes 2020 (OPTIC)* - Sasaki 2020 - PEARL study | - These three studies were excluded as they did not report data for outcome of interest (i.e. TTD or response) |
| - CML-202 - CML-203 | - Studies with omacetaxine (n=2) were included in the SLR but excluded from the MAIC because the intervention has limited approvals and does not represent worldwide standard of care in the third-line setting for CP-CML |

*Baseline characteristics (and associated outcomes) were not published separately for patients without the T315I mutation at baseline in OPTIC. Given that patients in ASCEMBL were randomized to asciminib or bosutinib, and bosutinib is ineffective in patients with T315I, the presence of this mutation mandated exclusion from the trial

#### Online Resource 4: Detailed Methodology of the Matching-Adjusted Indirect Treatment Comparison

In the context of unanchored indirect comparisons, the simplest approach to evaluate the relative treatment effect, based on non-randomized studies, is to compare the reported outcomes of interest from each study without adjusting for any between-study differences. This comparison is often referred to as a naive (unanchored) indirect comparison. Estimates resulting from a naive indirect comparison rely on the assumption that there is no imbalance between the studies with respect to prognostic factors nor treatment effect modifiers. However, this assumption is not defendable in most cases; that is, the treatment effect estimates are subject to a serious risk of bias.

It is possible to adjust for between-study differences, primarily in terms of the patient characteristics, to reduce the bias in the treatment effect estimates inherent in a naive indirect comparison. It is easier to justify an assumption of the conditional constancy of relative treatment effects, which implies the observed effect at some covariate value is the same in both populations. The most appropriate methodology to adjust for between-study differences depends on the availability of individual patient data (IPD) for each study. IPD for the comparators are often not available; therefore, these analyses tend to be limited to study-level aggregate data (AD) publications as identified from an SLR for the comparators (i.e., the PACE trial evaluating ponatinib). However, given the availability of IPD for the indexstudy, (i.e., the ASCEMBL trial evaluating asciminib), it was possible to adjust for between-study differences in the distribution of patient factors that may influence the outcome and/or treatment effects using a population-adjusted indirect comparison (PAIC).

MAIC reflects a method for PAIC, which uses the IPD from the index intervention (i.e., asciminib) and AD for the studies of comparators to weigh the IPD to better align with the comparator study population (DM 2018). The first step in conducting an MAIC is to match the index study with available IPD based on the eligibility criteria specified in the comparator study. This step involves removing patients from the index study if they do not fulfill the eligibility criteria of the comparator study. Initially, a logistic propensity score is then used to estimate weights for the IPD from the index trial so that the weighted mean baseline characteristics of the IPD match those published for the comparator study population. Guidelines for these methods have been developed by the Decision Support Unit commissioned by the National Institute for Health and Care Excellence (NICE) given that these methods are often used in the context of health technology assessments.(D 2016)

The estimation of these propensity weights is complicated by the lack of IPD in the comparator study; a modified likelihood re-weighting approach is employed that estimates weights from a logistic regression model:

| $\log\left( w_{i} \right)= \alpha_{0}+ {\boldsymbol{\alpha}_{1}^{T}\boldsymbol{X}}_{i}$ | (1) |
| --- | --- |

For each patient i, with covariates X_i_, in the index set. Standard regression techniques cannot be employed to generate these weights as the full distribution of covariates is not available for the comparator study. Following the NICE recommendations, the method of moments approach outlined by Signorovitch (2010) is used to balance the mean covariate values across populations. The weights are obtained by minimizing:

| $\sum_{i=1}^{N} exp({\boldsymbol{\alpha}_{1}^{T}\boldsymbol{X}}_{i})$ | (2) |
| --- | --- |

The weighting scheme is defined based only on the covariates and is therefore independent of the outcome. Although this implies that the weights can be used on any scale, all treatment comparisons must be conducted on the appropriate scale of the outcome, as the comparisons assume additivity.

The validity of a MAIC model depends upon the overlap between the IPD of the index study and the AD of the comparator study. When there is little overlap between the populations, the estimates are heavily influenced by relatively few individuals. Therefore, it is important to evaluate the distribution of the patient characteristics and the effect of the weighting to assess the appropriateness of the weights.

The weights are first rescaled relative to the unit weights of the original dataset based on the sample size (N), which facilitates the interpretation of the distribution of weights:

| $\tilde{w_{i}}= \frac{w_{i}N}{\sum_{i}^{N} w_{i}}$ | (3) |
| --- | --- |

Patients with rescaled weights greater than one provide more information when matched to the target population than when matched to the index population, and vice versa for patients with weights less than one. A measure of the extent of overlap is represented by the effective sample size (ESS). Signorovitch ([2010](#_ENREF_15)) suggest that the ESS of the pseudo-population formed by weighting the index study population can be approximated by:

| $ESS= \frac{{(\sum_{t=1}^{T} \sum_{i=1}^{N} w_{it})}^{2}}{\sum_{t=1}^{T} \sum_{i=1}^{N} w_{it}^{2}}$ | (4) |
| --- | --- |

ESS is an adjustment of the sample size that accounts for the weighting of the observations, and the resulting correlations between estimated responses. A large sample size is preferable to a small one, as a larger sample contains more information. For time-to-event data, if a Kaplan-Meier (KM) curve available in the comparator study, its data can be extracted by digitizing the curve and the IPD can be reconstructed using the Guyot algorithm (Guyot 2012). This reconstructed IPD could then be used to estimate relative treatment effects. A weighted Cox proportional hazards model could then be used to estimate the hazard ratio (HR) for TTD compared between the index treatment (i.e., asciminib) and the comparator treatment.

After the index trial has been adjusted to closely align with the comparator study’s population, the outcomes for patients receiving the index intervention (i.e., asciminib) can be estimated as if they were enrolled in the comparator study. An appropriate model is then used to estimate the outcome depending on the type of outcome. After estimating the outcomes for the population treated with the index intervention, a comparative treatment effect estimate between the index intervention and the comparator can then be calculated. A summary of methods and measure of treatment effects for different types of outcomes are presented in **Online Resource Table 3**.

Online Resource Table 3: Commonly used methods and treatment effects for different types of outcomes

| **Outcome** | **Method** | **Measure of Treatment Effect** | **Interpretation** |
| --- | --- | --- | --- |
| Continuous | Linear regression model | Mean difference | The average difference between the reference group and the comparator’s group. |
| Dichotomous | Logistic regression model | OR | The odds of an event occurring in the reference group compared to the odds of an event occurring in the comparator’s group. |
|  | Generalized linear model with log link | RR | The risk of an event occurring in the reference group compared to the risk of an event occurring in the comparator’s group. |
|  | Ratio of risks |  |  |
| Time-to-event | Cox proportional hazards model | HR | The risk of event occurring in a very small time interval for a patient in the reference group compared to a patient in the comparator’s group, given the patient survived up to a specific time. |

HR, hazard ratio; OR, odds ratio; RR, relative risk.

| Relative efficacy for asciminib versus comparator TKIs was determined for major molecular response (MMR) and complete cytogenic response (CCyR) rates by estimating the relative risks (RRs) and their 95% confidence intervals (CIs). The RR was calculated by taking the ratio of risks. That is, the risk of response in the reweighted patients who received the index intervention (i.e., asciminib) divided by the risk of response in the patients who received the comparator treatment: $\mathrm{RR}= \frac{(\frac{n_{a}}{N_{a}})}{(\frac{n_{c}}{N_{c}})}$ | (5) |
| --- | --- |

where $n_{a}$ is the number of patients who responded to the index intervention, $N_{a}$ is the total number of patients who received index intervention, $n_{c}$ is the number of patients who responded to the comparator treatment, and $N_{c}$is the total number of patients who received comparator treatment.

The lower and upper limits of the CI were then calculated by:

| $CI=\exp\left[ ln(RR)\pm z\sqrt{\frac{1}{n_{a}}-\frac{1}{N_{a}}+\frac{1}{n_{c}}-\frac{1}{N_{c}}} \right]$ | (6) |
| --- | --- |

where *z* is the Z-value (approximately 1.96 for 95% CIs

Relative efficacy for TTD is typically estimated by a hazard ratio (HR), which is calculated using as Cox proportional hazard model. This method requires that Kaplan-Meier (KM) curves are reported for the competing treatments, so that they can be leveraged to capture the data at every time point. However, none of the comparator trials reported a TTD KM curve; thus, it was not possible to calculate an indirect treatment effect estimate for TTD. Alternatively, the median TTD of the adjusted population receiving asciminib was compared to the median treatment duration reported for each of the comparators.

#### Online Resource 5: Range (min, max) of cumulative MMR, CCyR and median TTD across different MAIC scenarios

Online Resource Table 4: Range (min, max) of cumulative MMR, CCyR and median TTD across different MAIC scenarios

| Comparison | MMR (RR) | | CCyR | | Median time to response | |  |
| --- | --- | --- | --- | --- | --- | --- | --- |
|  | by 6 months | by 12 months | by 6 months | by 12 months | Time to MMR | Time to CCyR | Median TTD |
| Asciminib (N=103)* vs Ponatinib* (Cohort A, N=203) | (1.28, 1.56) | (1.27, 1.48) | (0.98, 1.18) | (0.87, 1.02) | xxxx | xxxx | - |
| Asciminib (N=103)* vs Ponatinib* (Cohort A+B, N=270) | - | - | - | - | - | - | xxxx |
| Asciminib (N=157) vs Nilotinib (N=39) | - | - | - | - | - | - | xxxx |
| Asciminib (N=103)* vs Nilotinib/Dasatinib (N=26) | - | - | (3.56, 3.88) | (1.91, 2.07) | - | - | - |
| Asciminib (N=157) vs Dasatinib (N=24) | (1.23, 1.45) | - | - | - | - | - | - |
| Asciminib (N=157) vs Dasatinib (N=34) | - | - | - | - | - | - | xxxx |

**Note**: PACE cohort A (N=203) includes patients on ≥3L CP-CML therapy; cohort A+B (N=270) includes 203 patients on ≥3L CP-CML therapy and 67 patients with T315I BCR::ABL1 mutation ≥1L CP-CML therapy.
*Since patients with CCyR at baseline were excluded in the PACE trial and Ibrahim et al, patients from ASCEMBL trial who either had CCyR at baseline (n=19) or if baseline CCyR data was missing (n=35) were similarly removed from the comparison to match the exclusion criteria.

MAIC, matching-adjusted indirect comparisons; MMR, major molecular response; CCyR, complete cytogenetic response; RR, risk ratio

#### Online Resource 6: Comparison of asciminib (excluding ponatinib pretreated patients) and ponatinib patients in achieving MMR/CCyR by 6 months and 12 months

Online Resource Table 5: Comparison of asciminib (excluding ponatinib pretreated patients) and ponatinib patients in achieving MMR/CCyR by 6 months and 12 months

|  | **MMR** | | | | | | **CCyR** | | | | |
| --- | --- | --- | --- | --- | --- | --- | --- | --- | --- | --- | --- |
| **Study** | **6 months** | | | **12 months** | | | **6 months** | | | **12 months** | |
|  | **%patients** | **RR  [95% CI]** | **%patients** | | **RR  [95% CI]** | **%patients** | | **RR  [95% CI]** | **%patients** | | **RR  [95% CI]** |
| ASCEMBL – Pre MAIC (n=90) | 31% | - | 39% | | - | 44% | | - | 50% | | - |
| ASCEMBL – Post MAIC (n=38) | 31% | 1.68  [1.1, 2.55] | 40% | | 1.72 [1.2, 2.45] | 39% | | 1.15  [0.84, 1.59] | 45% | | 1.03  [0.78, 1.36] |
| PACE cohort A (n=203) | 19% |  | 23% | |  | 34% | |  | 43% | |  |

Note: PACE cohort A (N=203) includes patients on ≥3L CP-CML therapy.
Ponatinib pretreated patients in the ASCEMBL trial were excluded from this analysis. Also, as patients with CCyR at baseline were excluded in the PACE trial, patients from ASCEMBL trial who either had CCyR at baseline (n=19) or if baseline CCyR data was missing (n=35) were similarly removed from the comparison to match the exclusion criteria.

MAIC, matching-adjusted indirect comparisons; MMR, major molecular response; CCyR, complete cytogenetic response, RR, risk ratio

#### Online Resource 7: Comparison of time to MMR and CCyR curves for asciminib (excluding ponatinib pretreated patients) and ponatinib

Online Resource Figure 2: Comparison of time to MMR and CCyR curves for asciminib (excluding ponatinib pretreated patients) and ponatinib

| 1. **MMR, Asciminib (excluding ponatinib pretreated patients; ASCEMBL) vs Ponatinib (PACE cohort A)**   **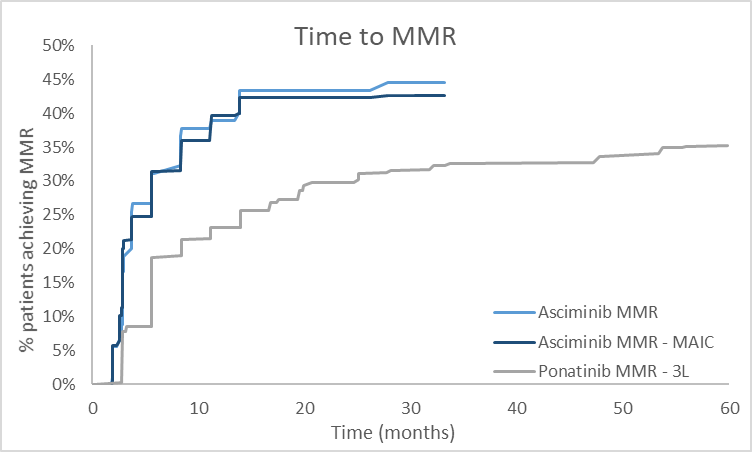** | 1. **CCyR, Asciminib (excluding ponatinib pretreated patients ASCEMBL) vs Ponatinib (PACE cohort A)**   **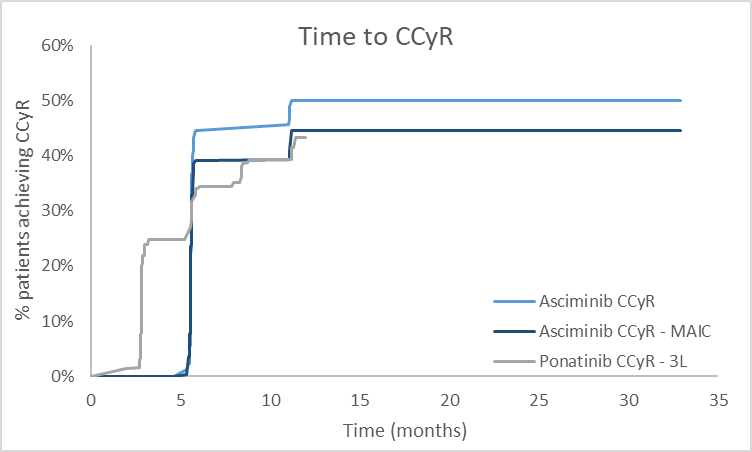** |
| --- | --- |

#### Online Resource 8: Naive comparison of reported safety outcomes

Due to numerous variables influencing the frequency of AEs, including the differences in definition and classification of AEs, adjusting the between-study differences as part of MAIC methodology was not feasible. Instead, the safety profile of asciminib was compared naively with the different comparators.

Most of the included studies reported the percentage of AE-related treatment discontinuations, whereas the proportion of patients with AEs, treatment-related AEs, and serious AEs were reported only for ASCEMBL, and PACE. Among the patients treated with asciminib, the proportion of treatment discontinuations due to an AE was 5.8% (follow-up: 6 months) compared with 41.2% with dasatinib (follow-up: not reported), 22.6% with ponatinib (follow-up: 12 months), and 10.3% with nilotinib (follow-up: 12 months). Among the included studies, asciminib had a better overall safety profile. A summary of the results of the naïve comparison of safety outcomes for this analysis is presented **Online Resource Table 6**.

Online Resource Table 6: Naive comparison of safety outcomes reported in the studies included in the analysis, n (%)

| **Study** | **Intervention** | **N** | **Any-cause AEs** | | **Treatment-related AEs** | | **Serious AEs** | **Treatment discontinuation due to AEs** | **Treatment-related deaths** |
| --- | --- | --- | --- | --- | --- | --- | --- | --- | --- |
|  |  |  | **Any grade** | **Grade 3/4** | **Any grade** | **Grade 3/4** |  |  |  |
| **ASCEMBL** | Asciminib | 156 | 140 (89.7) | 79 (50.6) | 99 (63.5) | 46 (29.5) | 2 (1.3) | 9 (5.8) | 2 (1.3) |
| **PACE^a^** | Ponatinib | 203 | 203 (100) | -- | -- | -- | 132 (65.5) | 46 (22.6) | -- |
| **Giles et al. 2010** | Nilotinib | 39 | -- | -- | -- | -- | -- | 4 (10.3) | -- |
| **Tan et al. 2019** | Dasatinib | 24 | -- | -- | -- | -- | -- | -- | -- |
| **Rossi et al. 2013** | Dasatinib | 82 | -- | -- | -- | -- | -- | 14 (41.2)^b^ | -- |
| **Ibrahim et al. 2010** | Nilotinib/dasatinib | 26 | -- | -- | -- | -- | -- | -- | -- |

^a^Outcomes reported are for the non-T315I mutated population; ^b^From the principal Khoury (2012) publication.

AE, adverse event.

#### References in supplementary material:

- Alessandra Iurlo, Mario Annunziata, Francesco Albano, Luigia Luciano, Raffaele Spadano, Anna Rita Scortechini, Massimiliano Bonifacio, Elisabetta Abruzzese, Monia Lunghi, Alessandra Malato, Nicola Di Renzo, Alfonso Piciocchi, Angela Pellegrino, Claudia Galimberti, Robin Foà, Massimo Breccia; Multicenter, Prospective and Retrospective Observational Cohort Study of Ponatinib in Patients with CML in Italy: Interim Analysis of the OITI Trial. Blood 2019; 134 (Supplement_1): 1652. doi: https://doi.org/10.1182/blood-2019-126098
- Carlo Gambacorti-Passerini, Camille N. Abboud, Bjorn T. Gjertsen, Tim H. Brümmendorf, B. Douglas Smith, Pilar Giraldo-Castellano, Ulla Olsson-Strömberg, Susanne Saussele, Nathalie Bardy-Bouxin, Andrea Viqueira, Eric Leip, Jocelyn M Leone, Gianantonio Rosti, Justin M. Watts, Frank Giles, Andreas Hochhaus, and BYOND Study Investigators. Primary results of the phase 4 BYOND study of bosutinib (BOS) for pretreated chronic phase (CP) chronic myeloid leukemia (CML). Journal of Clinical Oncology 2019 37:15_suppl, 7012-7012
- Chan O, Talati C, Isenalumhe L, Shams S, Nodzon L, Fradley M, Sweet K, Pinilla-Ibarz J. Side-effects profile and outcomes of ponatinib in the treatment of chronic myeloid leukemia. Blood advances. 2020 Feb 11;4(3):530-8.
- Chitanava T, Lomaia E, Shuvaev V, Martynkevich I, Fominykh M, Efremova E, Kersilova A, Poshivay AP, Koryagina E, Ilyina N, Dorofeeva N. CML-366: Baseline Cytogenetic Response Level Impact on Survival of Chronic Phase Chronic Myeloid Leukemia Patients Treated with Tyrosine Kinase Inhibitors as Third-Line Therapy: Real-World Data in Five Russian Centers. Clinical Lymphoma Myeloma and Leukemia. 2020 Sep 1;20:S242.
- Cortes J, Kantarjian HM, Richard-Carpentier G, Borthakur GM, Ravandi F, Naqvi K, Sasaki K, Kadia TM, Verstovsek S, Garcia-Manero G, DiNardo CD. Effectiveness of Bosutinib in Chronic Myeloid Leukemia (CML) Who Have Received Multi Tyrosine Kinase Inhibitors (TKIs). ASH, 2019.
- Cortes J, Lipton JH, Rea D, Digumarti R, Chuah C, Nanda N, Benichou AC, Craig AR, Michallet M, Nicolini FE, Kantarjian H. Phase 2 study of subcutaneous omacetaxine mepesuccinate after TKI failure in patients with chronic-phase CML with T315I mutation. Blood, The Journal of the American Society of Hematology. 2012 Sep 27;120(13):2573-80.
- Cortes JE, Apperley J, Hochhaus A, Mauro MJ, Rousselot P, Sacha T, Talpaz M, Chuah C, Lipton JH, Deininger MW, Schiffer CA. Outcome by mutation status and line of treatment in Optic, a dose-ranging study of 3 starting doses of ponatinib in patients with CP-CML. InBlood 2020 Nov 5 (Vol. 136). 2021 L ST NW, Suite 900, Washington, Dc 20036 Usa: Amer Soc Hematology.
- Cortes JE, Kantarjian H, Shah NP, Bixby D, Mauro MJ, Flinn I, O'Hare T, Hu S, Narasimhan NI, Rivera VM, Clackson T. Ponatinib in refractory Philadelphia chromosome–positive leukemias. New England Journal of Medicine. 2012 Nov 29;367(22):2075-88.
- D, P. (2016). NICE DSU Technical Support Document 18: Methods for population-adjusted indirect comparisons in submissions to NICE, NICE.
- Deininger MW, Réa D, Lang F, Kim DW, Cortes JE, Hughes TP, Minami H, Breccia M, DeAngelo DJ, Hochhaus A, Talpaz M. Efficacy and Safety of Asciminib, a Specific Allosteric BCR-ABL1 Inhibitor Targeting the Myristoyl-Binding Site, in Patients with Chronic Myeloid Leukemia (CML) Carrying the T315I Mutation. Clinical Lymphoma, Myeloma and Leukemia. 2019 Sep 1;19:S289-90.
- Devos T, Havelange V, Theunissen K, Meers S, Benghiat FS, Gadisseur A, Vanstraelen G, Vellemans H, Bailly B, Granacher N, Lewalle P. Clinical outcomes in patients with Philadelphia chromosome-positive leukemia treated with ponatinib in routine clinical practice—data from a Belgian registry. Annals of hematology. 2021 May 4:1-0
- DM, P. (2018). "Methods for Population-Adjusted Indirect Comparisons in Health Technology Appraisal." Medical decision making : an international journal of the Society for Medical Decision Making 38(2): 200-211.
- E.Signorovitch, J. (2010). "Comparative Effectiveness Without Head-to-Head Trials A Method for Matching-Adjusted Indirect Comparisons Applied to Psoriasis Treatment with Adalimumab or Etanercept." Pharmacoeconomics 28(10): 935-945.
- García-Gutiérrez V, Milojkovic D, Hernandez-Boluda JC, Claudiani S, Mateos ML, Casado-Montero LF, González G, Jimenez-Velasco A, Boque C, Martinez-Trillos A, Vázquez IM. Safety and efficacy of bosutinib in fourth-line therapy of chronic myeloid leukemia patients. Annals of hematology. 2019 Feb;98(2):321-30.
- Garg RJ, Kantarjian H, O'Brien S, Quintas-Cardama A, Faderl S, Estrov Z, et al. The use of nilotinib or dasatinib after failure to 2 prior tyrosine kinase inhibitors: long-term follow-up. Blood. 2009;114(20):4361-8.
- Guerci-Bresler A, Turhan A, Rousselot P, Gabriel E, Coiteux V, Berger M, Cayssials E, Huguet F. French Real–Life Observational Study “Topase” Evaluating Safety And Efficacy Of Ponatinib Confirms Induction Of Deep Molecular Responses In 110 Resistant Or Intolerant CML Patients. EHA2021 Virtual Congress 2021 Jun 9.
- Gugliotta G, Annunziata M, Capodanno I, Rapezzi D, Attolico I, Vincelli ID, Tiribelli M, Malato A, Pizzuti M, Accurso V, Bonifacio M, Galimberti S, Loglisci G, Abruzzese E, Bocchia M, Gozzini A, Bergamaschi M, Miggiano MC, Tafuri A, Sanpaolo G, Binotto G, Castagnetti F, Specchia G, Di Raimondo F, Cavo M, Rosti G, Foà R, Saglio, Breccia M, and Stagno F. Sequential Treatments in Chronic Phase Chronic Myeloid Leukemia (CML) Patients without Optimal Response after Frontline Nilotinib or Dasatinib: An Italian CML Campus Study. ASH, 2020.
- Guyot, P., A. E. Ades, M. J. N. M. Ouwens and N. J. Welton (2012). "Enhanced secondary analysis of survival data: reconstructing the data from published Kaplan-Meier survival curves." BMC Medical Research Methodology 12(1): 9.
- Heiblig M, Rea D, Chrétien ML, Charbonnier A, Rousselot P, Coiteux V, Escoffre-Barbe M, Dubruille V, Huguet F, Cayssials E, Hermet E. Ponatinib evaluation and safety in real-life chronic myelogenous leukemia patients failing more than two tyrosine kinase inhibitors: the PEARL observational study. Experimental hematology. 2018 Nov 1;67:41-8
- J Valentin Garcia-Gutierrez, Begoña Maestro, Luis Felipe Casado, Manuel Perez-Encinas, Isabel Massague, Raquel de Paz, Santiago Osorio, Joaquin Martinez, Guiomar Bautista, Pilar Giraldo, Carmen Burgaleta, Maria Jesús Peñarrubia, Maria José Requena, Carmen Calle, Jose Ángel Hernández-Rivas, Pilar Cano, Juan Luis Steegmann; Outcomes of Chronic Myeloid Leukemia (CML) Patients Who Stopped Second Generation Tyrosine Kinase Inhibitors (2GTKIs) As Second Line Treatment. Results of the CML Spanish National Registry (RELMC). Blood 2012; 120 (21): 3764. doi: https://doi.org/10.1182/blood.V120.21.3764.3764
- Jiang Q, Huang X, Chen Z, Niu Q, Shi D, Li Z, Hou Y, Hu Y, Li W, Liu X, Xu N. Novel BCR-ABL1 tyrosine kinase inhibitor (TKI) HQP1351 (olverembatinib) is efficacious and well tolerated in patients with T315I-mutated chronic myeloid leukemia (CML): results of pivotal (phase II) trials. InBlood 2020 Nov 5 (Vol. 136). 2021 L ST NW, Suite 900, Washington, Dc 20036 Usa: Amer Soc Hematology.
- Khoury HJ, Cortes JE, Kantarjian HM, Gambacorti-Passerini C, Baccarani M, Kim DW, Zaritskey A, Countouriotis A, Besson N, Leip E, Kelly V. Bosutinib is active in chronic phase chronic myeloid leukemia after imatinib and dasatinib and/or nilotinib therapy failure. Blood, The Journal of the American Society of Hematology. 2012 Apr 12;119(15):3403-12.
- Koji Sasaki, Elias Jabbour, Ghayas C. Issa, Guillermo Garcia-Manero, Tapan M. Kadia, William G. Wierda, Musa Yilmaz, Courtney D. DiNardo, Jeffrey Skinner, Maria Khouri, Naveen Pemmaraju, Patrice Nasnas, Sherry A. Pierce, Jorge E. Cortes, Hagop M. Kantarjian; Outcomes of Patients with Chronic Myeloid Leukemia Treated with Third-Line Tyrosine Kinase Inhibitors. Blood 2020; 136 (Supplement 1): 25–26. doi: https://doi.org/10.1182/blood-2020-142954
- Latagliata R, Attolico I, Trawinska MM, Capodanno I, Annunziata M, Elena C, Luciano L, Crugnola M, Bergamaschi M, Bonifacio M, Baratè C. Bosutinib in the real‐life treatment of chronic myeloid leukemia patients aged> 65 years resistant/intolerant to previous tyrosine‐kinase inhibitors. Hematological Oncology. 2021 Feb 22.
- Lee SE, Choi SY, Kim SH, Jang EJ, Bang JH, Byeun JY, Park JE, Jeon HR, Oh YJ, Yahng SA, Cho BS. Prognostic factors for outcomes of allogeneic stem cell transplantation in chronic phase chronic myeloid leukemia in the era of tyrosine kinase inhibitors. Hematology. 2014 Mar 1;19(2):63-72.
- Luna A, Estrada N, Boque C, Xicoy B, Giraldo P, Angona A, Alvarez-Larran A, Sanchez-Guijo F, Ramirez MJ, Alonso-Dominguez JM, Mora E. Safety and Efficacy Profile of Asciminib As Treatment in Chronic Myeloid Leukemia Patients after Several Tyrosine-Kinase Inhibitors Failure. In Blood 2020 Nov 5 (Vol. 136). 2021 L ST NW, Suite 900, Washington, DC 20036 USA: Amer Soc Hematology.
- Mahesh Swaminathan, Hagop M. Kantarjian, Koji Sasaki, Farhad Ravandi, Gautam Borthakur, Tapan M. Kadia, Guillermo Garcia-Manero, Courtney D. DiNardo, Marina Y. Konopleva, Zeev E. Estrov, Naveen Pemmaraju, Naval G. Daver, William G. Wierda, Alessandra Ferrajoli, Sherry A. Pierce, MD Emogene Dellasala, Elias J. Jabbour, Jorge E. Cortes; Efficacy of Ponatinib after Multiple Lines of Therapy for Chronic Myeloid Leukemia. Blood 2018; 132 (Supplement 1): 3013. doi: https://doi.org/10.1182/blood-2018-99-119914
- Maliha Khan, Hagop M. Kantarjian, Jing Ning, Mary Akosile, Wen Li, Gautam Borthakur, Nitin Jain, Courtney D. DiNardo, William G Wierda, Farhad Ravandi, Guillermo Garcia-Manero, Marina Konopleva, Tapan Kadia, Naveen Pemmaraju, Philip A Thompson, Naval Daver, Elias J. Jabbour, Jorge E. Cortes; Response and Outcomes of Third-Line Tyrosine Kinase Inhibitor Therapy on Patients with Chronic Phase Chronic Myeloid Leukemia. Blood 2017; 130 (Supplement 1): 2882. doi: https://doi.org/10.1182/blood.V130.Suppl_1.2882.2882
- Mario Tiribelli, Elisabetta Abruzzese, Isabella Capodanno, Federica Sorà, Elena Trabacchi, Alessandra Iurlo, Luigia Luciano, Gianni Binotto, Massimiliano Bonifacio, Mario Annunziata, Monica Crugnola, Renato Fanin; Efficacy and Safety of Bosutinib in Chronic Phase CML Patients Developing Pleural Effusion Under Dasatinib Therapy. Blood 2018; 132 (Supplement 1): 5439. doi: https://doi.org/10.1182/blood-2018-99-113537
- Ongoren S, Eskazan AE, Suzan V, Savci S, Erdogan Ozunal I, Berk S, Yalniz FF, Elverdi T, Salihoglu A, Erbilgin Y, Iseri SA. Third-line treatment with second-generation tyrosine kinase inhibitors (dasatinib or nilotinib) in patients with chronic myeloid leukemia after two prior TKIs: real-life data on a single center experience along with the review of the literature. Hematology. 2018 Apr 21;23(4):212-20.
- Qian Jiang, Xiaojun Huang, Zi Chen, Qian Niu, Lichuang Men, Hengbang Wang, Jiao JI, Bo Huang, Dayu Shi, Ting Zhao, Yue Hou, Dajun Yang, Yifan Zhai; An Updated Safety and Efficacy Results of Phase 1 Study of HQP1351, a Novel 3rd Generation of BCR-ABL Tyrosine Kinase Inhibitor (TKI), in Patients with TKI Resistant Chronic Myeloid Leukemia. Blood 2019; 134 (Supplement_1): 493. doi: https://doi.org/10.1182/blood-2019-124295
- Ribeiro BF, Miranda E, Albuquerque DM, Delamain MT, Oliveira-Duarte G, Almeida MH, Vergílio B, Silveira RA, Oliveira-Duarte V, Lorand-Metze I, Souza CA. Treatment with dasatinib or nilotinib in chronic myeloid leukemia patients who failed to respond to two previously administered tyrosine kinase inhibitors-a single center experience. Clinics. 2015;70:550-5.
- Takahashi N, Nakaseko C, Kobayashi Y, Miyamura K, Ono C, Koide Y, Fujii Y, Ohnishi K. Long-term treatment with bosutinib in a phase 1/2 study in Japanese chronic myeloid leukemia patients resistant/intolerant to prior tyrosine kinase inhibitor treatment. International journal of hematology. 2017 Sep;106(3):398-410.
- Tojo A, Kyo T, Yamamoto K, Nakamae H, Takahashi N, Kobayashi Y, Tauchi T, Okamoto S, Miyamura K, Hatake K, Iwasaki H. Ponatinib in Japanese patients with Philadelphia chromosome-positive leukemia, a phase 1/2 study. International journal of hematology. 2017 Sep;106(3):385-97.
- Turkina AG, Shukhov OA, Chelysheva EY, Nemchenko IS, Petrova A, Bykova A, Zaritskey A, Lomaia E, Siordiya N, Vinogradova O, Shuvaev V. Updated results from the ongoing phase I study of PF-114 mesylate in patients with CML with failure of prior TKI therapy. HemaSphere. 2018;2(S1):139-40.
